# Variant Curation of the Largest Compendium of *FOXL2* Coding and Noncoding Sequence and Structural Variants in BPES

**DOI:** 10.1155/humu/8478740

**Published:** 2026-05-06

**Authors:** Charlotte Matton, Julie Van De Velde, Marieke De Bruyne, Stijn Van De Sompele, Sally Hooghe, Hannes Syryn, Miriam Bauwens, Eva D′haene, Annelies Dheedene, Martine Cools, Shoko Komatsuzaki, Ewelina Preizner-Rzucidło, Alison Ross, Christine Armstrong, Wendy Watkins, Andrew Shelling, Andrea L. Vincent, Catherine Cassiman, Sascha Vermeer, David J. Bunyan, Hannah Verdin, Elfride De Baere

**Affiliations:** ^1^ Department of Biomolecular Medicine, Ghent University, Ghent, Belgium, ugent.be; ^2^ Center for Medical Genetics Ghent, Ghent University Hospital, Ghent, Belgium, uzgent.be; ^3^ Department of Pediatric Endocrinology, Ghent University Hospital, Ghent, Belgium, uzgent.be; ^4^ Department of Internal Medicine and Pediatrics, Ghent University, Ghent, Belgium, ugent.be; ^5^ Institute of Human Genetics, University of Würzburg, Biozentrum Am Hubland, Würzburg, Germany, uni-wuerzburg.de; ^6^ Department of Molecular Genetics, Institute of Pediatrics, Jagiellonian University Medical College, Krakow, Poland, cm-uj.krakow.pl; ^7^ North of Scotland Regional Genetics Service, Laboratory Genetics, Aberdeen Royal Infirmary, Aberdeen, UK, nhsgrampian.org; ^8^ Department of Obstetrics and Gynaecology, Faculty of Medical and Health Sciences, The University of Auckland, Auckland, New Zealand, auckland.ac.nz; ^9^ Centre for Cancer Research, Faculty of Medical and Health Sciences, The University of Auckland, Auckland, New Zealand, auckland.ac.nz; ^10^ Department of Ophthalmology, Faculty of Medical and Health Sciences, The University of Auckland, Auckland, New Zealand, auckland.ac.nz; ^11^ Department of Ophthalmology, Leuven University Hospital, Louvain, Belgium; ^12^ Centre of Human Genetics, University Hospitals Leuven, Louvain, Belgium, uzleuven.be; ^13^ Wessex Regional Genomics Laboratory, Salisbury District Hospital, Salisbury, Wiltshire, UK, wrgl.org.uk; ^14^ Faculty of Medicine, University of Southampton, Southampton, Hampshire, UK, southampton.ac.uk

## Abstract

Heterozygous *FOXL2* (non)coding sequence and structural variants (SVs) lead to blepharophimosis, ptosis and epicanthus inversus syndrome (BPES), a rare, autosomal dominant developmental disorder characterized by a completely penetrant eyelid malformation and incompletely penetrant primary ovarian insufficiency (POI). We collected variants from our in‐house database, generated via clinical genetic testing and downstream research testing in the Center for Medical Genetics Ghent, Belgium (2001–2024) and via literature and other resources in the same period. All retrieved variants were categorized using ACMG/AMP classifications to increase the knowledge of pathogenicity. We collected 413 unique genetic defects of the *FOXL2* region, including 76 novel variants, in 864 index patients. Of these, 87% of patients were identified with a coding *FOXL2* sequence variant. The polyalanine tract is a known mutational hotspot of *FOXL2*, illustrated here by the high percentage of pathogenic polyalanine expansions (24%). Furthermore, the molecular spectrum in typical BPES index patients is characterized by 8% coding deletions and 3% deletions located up‐ and downstream of *FOXL2*. The remaining 2% carry translocations along with chromosomal rearrangements of 3q23. This uniform and structured reclassification, incorporating the largest dataset of variants implicated in *FOXL2*‐associated disease so far, will improve both the diagnosis as well as genetic counselling for individuals with BPES.

## 1. Introduction

*FOXL2* is a 2.9‐kb single‐exon gene located on the long arm of chromosome 3 (3q22.3). The gene encodes a 376‐amino‐acid transcription factor with a highly conserved DNA‐binding forkhead domain. In addition to this forkhead domain, a 14‐amino‐acid polyalanine tract can be distinguished, of which the function remains largely unknown (Figure 1a) [3–5].

FIGURE 1(a) The FOXL2 protein comprises 376 amino acids and contains two key domains: the forkhead domain in blue (p.54–164) and the polyalanine tract in red (p.221–234), adapted from Beysen *et al*. [1]. (b) The transcription factor FOXL2 is essential for ovarian development. At 5–6 weeks of gestation, the bipotential gonad differentiates towards either testis or ovaries, depending on the chromosomal sex. This process is tightly regulated by antagonistic interactions between key factors. FOXL2 inhibits SOX9, thereby repressing the protestis pathway and, in concert with other regulators, promoting ovarian differentiation. In adulthood, FOXL2 remains crucial for ovarian maintenance and function. Figure adapted from Tucker *et al*. [2].

**Figure:**
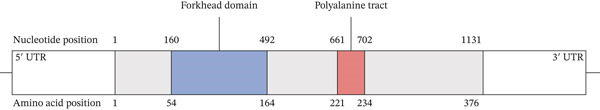
(a)

**Figure:**
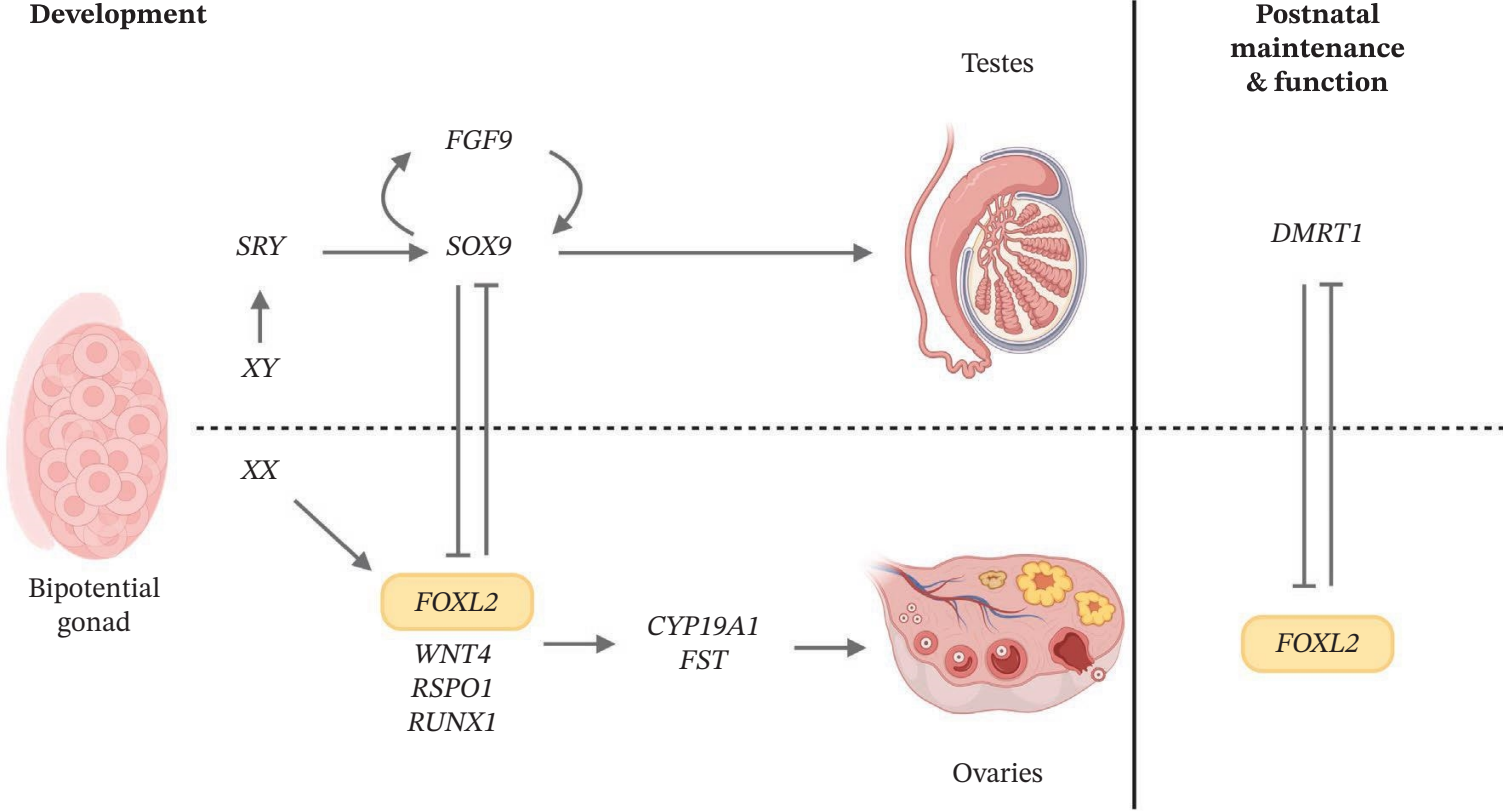
(b)

The FOXL2 transcription factor, like other members of the forkhead transcription factor family, is involved in key developmental processes and requires tightly controlled spatiotemporal gene regulation [6]. During embryonic development, FOXL2 is expressed in the developing periocular mesenchyme, where it activates *α* smooth muscle actin (*α*‐SMA), facilitating the formation of the levator smooth muscle [7]. In developing ovaries, FOXL2 functions alongside other profemale factors, promoting the upregulation of ovarian‐specific genes while simultaneously suppressing testis‐specific factors such as SOX9 [2, 8, 9] (Figure 1b). Postnatally, *FOXL2* is expressed in the granulosa cells (i.e., supporting cells of the oocyte), pituitary gland and uterus [2, 10–13]. In adult granulosa cells, FOXL2 is critical for ovarian maintenance and folliculogenesis, ensuring the integrity of ovarian function throughout reproductive life [14] (Figure 1b). In line with other highly conserved transcription factor genes, impairment of *FOXL2* results in developmental disease and cancer.

Heterozygous germline variants in *FOXL2* are known to cause blepharophimosis, ptosis and epicanthus inversus syndrome (BPES; OMIM #110100), a rare, autosomal dominant, developmental disorder that can arise *de novo* or be inherited [4, 5, 15]. BPES is characterized by a fully penetrant eyelid malformation and an incompletely penetrant and variable primary ovarian insufficiency (POI). The eyelid malformation is described according to four typical features including (1) horizontal (blepharophimosis) and (2) vertical (ptosis) narrowing of the palpebral fissures, (3) a skin fold running inwards and upwards from the lower eyelid (epicanthus inversus) and (4) a lateral displacement of the inner canthi (telecanthus). POI is defined as the absence of menses for over 4 months before the age of 40, resulting in early menopause and female subfertility or infertility due to impaired folliculogenesis in adult granulosa cells. BPES is associated with additional features including thick eyebrows, displacement of the lacrimal puncta, nasolacrimal drainage problems, a broad nasal bridge and low‐set ears [11, 15, 16].

At the somatic level, *FOXL2* variants are strongly implicated in adult‐type granulosa cell tumours, with a recurrent missense variant c.402C > G (p.Cys134Trp) detected in approximately 90%–95% of cases [17].

In this study, we compiled a large collection of in‐house variants identified through clinical and research genetic testing at our centre (Center for Medical Genetics Ghent, Ghent University Hospital, Ghent, Belgium) and elsewhere (Wessex Regional Genomics Laboratory, Salisbury District Hospital, Salisbury, United Kingdom; Institute of Human Genetics, University of Würzburg, Würzburg, Germany; Laboratory of Cytogenetics and Molecular Genetics, University Children′s Hospital, Krakow, Poland; Clinical Genetics Centre, Aberdeen Royal Infirmary, Aberdeen, United Kingdom; and Centre of Human Genetics, University Hospital Leuven, Louvain, Belgium) in index patients with a typical BPES phenotype. This dataset was complemented with previously published *FOXL2* sequence and structural variants (SVs) affecting the *FOXL2* region, all identified in patients with typical BPES. Together, this resulted in a comprehensive compendium of 413 distinct heterozygous constitutional *FOXL2* variants, ranging from coding sequence changes and copy number variants (CNVs) to translocations and noncoding CNVs disrupting putative *cis*‐regulatory elements (CREs). All sequence variants were subsequently classified according to the most recent American College of Medical Genetics and Genomics and the Association for Molecular Pathology (ACMG‐AMP) standard guidelines. Notably, this large study enabled us to expand the variant spectrum with 76 novel *FOXL2* genetic defects.

## 2. Material and Methods

### 2.1. Literature Search on Patients With BPES and a Confirmed *FOXL2* Genetic Defect

We collected publications up to December 2024 that reported typical BPES patients with a genetically confirmed heterozygous *FOXL2* variant, using the following PubMed search terms: ‘*FOXL2*’ [All Fields] AND (‘mutation’ [All Fields] OR ‘variant’ [All Fields] OR ‘translocation’ [All Fields] OR ‘deletion’ [All Fields]). The literature search was extended via Google Scholar to not exclude publications that were not listed in PubMed. Moreover, we mined the ClinVar and Leiden Open‐source Variation Database (LOVD) databases to include variants that were only submitted to the databases. Likewise, to ensure that unpublished SVs were not overlooked, the Genomics England and Decipher databases were consulted.

For all included variants, variant annotations, gnomAD allele frequencies (gnomAD V4.1.0), disease phenotype and patient information (including age and sex) were collected, if available. Duplicates were excluded from the variant collection based on overlap in patient information and author names of the included publications. In addition, studies for which the full text was unavailable or not accessible through the Ghent University Library, or not available in English, were excluded.

Although a comprehensive search was performed, we cannot exclude that certain *FOXL2* variants associated with BPES were missed due to the limitations of literature search tools (e.g., *FOXL2* variants solely mentioned in publications′ supporting information might have been overlooked).

### 2.2. Unreported Patients With BPES and a Confirmed Molecular Cause From the In‐House Patient Cohort

In addition to variants obtained from literature and public databases, *FOXL2* variants identified through clinical genetic testing at the Center for Medical Genetics Ghent (Ghent University Hospital, Ghent, Belgium), Wessex Regional Genomics Laboratory (Salisbury District Hospital, Salisbury, United Kingdom), Institute of Human Genetics (University of Würzburg, Würzburg, Germany), Laboratory of Cytogenetics and Molecular Genetics (University Children′s Hospital, Krakow, Poland), Clinical Genetics Centre (Aberdeen Royal Infirmary, Aberdeen, United Kingdom) and Centre of Human Genetics (University Hospital Leuven, Louvain, Belgium) were included. Given the specificity of the BPES phenotype and *FOXL2* being the only known disease locus for this rare syndrome, Sanger sequencing was performed across the *FOXL2* open reading frame in all cases with BPES. The initial polymerase chain reaction (PCR) to amplify the region of interest was performed using three overlapping primers pairs (Supporting Information 4: Table S4) and Kapa2G Robust Master Mix (2x, Kapa Biosystems, MA, United States), followed by Sanger sequencing using the BrilliantDye kit (V3.1, NimaGent, Nijmegen, the Netherlands) and ran on an ABI3730XL DNA analyzer (Applied Biosystems, MA, United States). Due to the GC‐rich nature of the *FOXL2* region, 5% dimethyl sulfoxide (DMSO) was added to increase sequencing signal intensity.

Multiplex ligation‐dependent probe amplification (MLPA) was performed using a commercially available probe mix (P054, MRC Holland, Amsterdam, the Netherlands) to detect CNVs. The MLPA kit contains three probes covering the coding *FOXL2* region, four probes covering the *ATR* region (Exon 1, 4, 22 and 47) and five probes overlapping with the *PISRT1* region, a long noncoding RNA (lncRNA) located in the regulatory region upstream of *FOXL2*. Apart from deletion identification itself, it is also essential to size the deletion, which was previously done using targeted arrays and qPCR [18–20].

### 2.3. Nomenclature

Variant annotations in the *FOXL2* coding region were (re)assessed using Alamut (GR Ch38) for coding DNA (c.), protein (p.) and genome (g.) nomenclature. When necessary, annotations were revised to align with the latest Human Genome Variation Society (HGVS) nomenclature guidelines (http://hgvs-nomenclature.org).

### 2.4. ACMG/AMP Variant Pathogenicity Classification

All collected coding *FOXL2* variants were (re)classified using an in‐house variant classification tool (VCT) (Center for Medical Genetics Ghent, Ghent University Hospital, Ghent, Belgium), developed in accordance with ACMG/AMP guidelines described by Richards *et al.* and Tavtigian *et al.* [21, 22]. The VCT categorizes variants into five classes based on their calculated *p* value, namely benign (*p* < 0.001), likely benign (0.001 ≤ *p* < 0.1), unknown significance (VUS) (0.1 ≤ *p* < 0.9), likely pathogenic (0.9 ≤ *p* < 0.99) or pathogenic (*p* ≥ 0.99).

### 2.5. LOVD and ClinVar Submission

Following the (re)classification of all coding *FOXL2* sequence variants, they were uploaded to the LOVD and ClinVar database to ensure comprehensive documentation of all collected *FOXL2* variants. When available, patient information and *in silico* predictions were also included.

This work was conducted according to the tenets of the Declaration of Helsinki and was approved by the data access committee of Ghent University Hospital.

## 3. Results

### 3.1. Collection of BPES Index Cases and Unique *FOXL2* Genetic Defects

Through a comprehensive literature and database search, we collected 497 index cases with a typical BPES phenotype that have been reported previously in international, peer‐reviewed literature and curated databases, including ClinVar, Genomics England and DECIPHER [4, 5, 15, 18–20, 23–117]. In addition, we report 367 BPES index patients with a genetic defect of the *FOXL2* region identified via routine diagnostic pipelines, resulting in a total collection of 864 index cases included in this study (Table 1 and 2, Supporting Information 1–3: Tables S1–S3). This large BPES patient cohort revealed 413 unique *FOXL2* genetic defects, both in the coding and noncoding regions of *FOXL2*. Notably, we report 76 novel variants associated with a typical BPES phenotype in this compendium (Table 1 and 2).

**TABLE 1:** (A) We identified 54 novel *FOXL2* coding variants, including missense, nonsense, frameshift and in‐frame indels, in this study. All variants are annotated with their corresponding ACMG/AMP variant classifications.

A. Novel coding *FOXL2* variants				
DNA variant	Predicted protein effect	ACMG‐AMP classification		Phenotype
c.75_78del	p.(Lys25Asnfs∗124)	LP	PM2, PP4_PM, PVS1_PS1, PP1	BPES
c.119del	p.(Gly40Valfs∗110)	LP	PM2_PP, PP4_PM, PVS1_PS1	BPES
c.136_137del	p.(Pro46Glyfs∗49)	P	PM2, PP4_PM, PVS1_PS1, PP1	BPES
c.328del	p.(Glu110Serfs∗40)	LP	PM2, PP4_PM, PVS1_PS1	BPES
c.395del	p.(Pro132Argfs∗18)	LP	PM2, PP4_PM, PVS1_PS1, PP1	BPES
c.467_473del	p.(Pro156Argfs∗113)	P	PM2, PP4_PM, PVS1_PS1, PS2	BPES
c.495dup	p.(Leu166Alafs∗73)	LP	PM2, PP4_PM, PVS1_PS1	BPES
c.501del	p.(Phe167Leufs∗104)	P	PM2, PP4_PM, PVS1_PS1, PM6	BPES
c.593del	p.(Gly198Alafs∗73)	LP	PM2, PP4_PP, PVS1_PS1	BPES
c.675_705del	p.(Ala226Profs∗35)	LP	PM2, PP4_PM, PVS1_PS1	BPES
c.735del	p.(Lys246Argfs∗25)	LP	PM2, PP4_PM, PVS1_PS2, PP1	BPES
c.749del	p.(Gly250Alafs∗21)	P	PM2, PP4_PM, PVS1_PS2, PS2	BPES
c.770dup	p.(Tyr258Valfs∗276)	P	PM2, PP4_PM, PVS1_PS2, PS2	BPES
c.806dup	p.(Val270Argfs∗264)	LP	PM2, PP4_PM, PVS1_PS2	BPES with POI
c.839del	p.(Pro280Argfs∗76)	LP	PM2, PP4_PM, PVS1_PS2	BPES
c.846dup	p.(Ala283Argfs∗251)	LP	PM2, PP4_PM, PVS1_PS2	BPES
c.858_873dup	p.(Pro292Serfs∗247)	LP	PM2, PP4_PM, PVS1_PS2	BPES
c.866_867dup	p.(Pro290Thrfs∗67)	LP	PM2, PP4_PM, PVS1_PS2	BPES
c.871_895del	p.(His291Cysfs∗57)	LP	PM2, PP4_PM, PVS1_PS2	BPES
c.965_978dup	p.(Pro327Serfs∗34)	LP	PM2, PP4_PM, PVS1_PS2	BPES
c.1078del	p.(Tyr360Thrfs∗91)	LP	PM2, PP4_PM, PVS1_PM	BPES
c.1084del	p.(Asp362Thrfs∗89)	LP	PM2, PP4_PM, PVS1_PM	BPES
c.19G > T	p.(Glu7∗)	LP	PM2, PP4_PM, PVS1_PS1	BPES
c.177C > G	p.(Tyr59∗)	LP	PM2, PP4_PM, PVS1_PS1	BPES
c.249C > A	p.(Tyr83∗)	LP	PM2, PP4_PM, PVS1_PS1	BPES
c.328G > T	p.(Glu110∗)	LP	PM2, PP4_PM, PVS1_PS1	BPES
c.331_332insA	p.(Cys111∗)	LP	PM2, PP4_PM, PVS1_PS1	BPES
c.403G > T	p.(Glu135∗)	LP	PM2, PP4_PM, PVS1_PS1, PM6	BPES with POI
c.528C > A	p.(Cys176∗)	LP	PM2, PP4_PM, PVS1_PS1	BPES
c.564C > G	p.(Tyr188∗)	LP	PM2, PP4_PM, PVS1_PS1	BPES
c.582C > A	p.(Tyr194∗)	LP	PM2, PP4_PM, PVS1_PS1	BPES
c.622C > T	p.(Gln208∗)	LP	PM2, PP4_PP, PVS1_PS1	BPES
c.736A > T	p.(Lys246∗)	LP	PM2, PP4_PM, PVS1_PS2	BPES
c.765C > A	p.(Tyr255∗)	P	PM2, PP4_PP, PVS1_PS2, PS2	BPES
c.163C > T	p.(Pro55Ser)	LP	PM2, PP4_PM, PP3, PM1_PP	BPES
c.167C > A	p.(Pro56Gln)	LP	PM2, PP4_PM, PP3, PM1_PP	BPES with mental retardation
c.175 T > C	p.(Tyr59His)	LP	PM2, PP4_PM, PP3, PM1_PP	BPES with POI
c.184C > G	p.(Leu62Val)	LP	PM2, PP4_PM, PP3, PM1_PP	BPES
c.196G > A	p.(Ala66Thr)	LP	PM2, PP4_PM, PP3, PM5_PM, PM1_PP	BPES
c.238A > T	p.(Ile80Phe)	LP	PM2, PP4_PM, PP3, PM1	BPES
c.271 T > C	p.(Tyr91His)	P	PM2, PP4_PM, PP3, PM1, PS2	BPES
c.272A > G	p.(Tyr91Cys)	LP	PM2, PP4_PM, PP3, PM1_PP	BPES
c.298A > C	p.(Asn100His)	LP	PM2, PP4_PM, PP3, PM1, PP1	BPES
c.300 T > A	p.(Asn100Lys)	LP	PM2, PP4_PM, PP3, PM1_PP, PP1	BPES
c.308G > C	p.(Arg103Pro)	LP	PM2, PP4_PM, PP3, PM5_PM, PM1_PP	BPES
c.334 T > A	p.(Phe112Ile)	LP	PM2, PP4_PM, PP3, PM1	BPES
c.377A > T	p.(Asn126Ile)	LP	PM2, PP4_PM, PP3, PM1_PP	BPES
c.142_173delinsGCGCT	p.(Lys48_Ser58delinsAlaLeu)	LP	PM2, PP4_PM, PM4_PM, PM6	BPES
c.191_233delins43	p.(Ala64_Ser78delins15)	LP	PM2, PP4_PM, PM4_PM	BPES
c.214_216del	p.(Glu72del)	LP	PM2, PS4_PP, PP4_PM, PM4_PP, PM6	BPES
c.666_698dup	p.(Ala224_Ala234dup)	P	PM2, PP4_PM, PM4_PM, PS3, PM6	BPES
c.701_702ins30	p.(Ala225_Ala234dup)	LP	PM2, PP4_PM, PM4_PM	BPES
c.1130G > C	p.(∗377Serext∗31)	LP	PM2, PS4_PP, PP4_PM, PM4_PM	BPES with POI
c.1331A > G	p.(∗377Trpext∗31)	LP	PM2, PP4_PM, PM4_PM, PM6	BPES

**TABLE 2:** (B) We identified 22 SVs affecting the *FOXL2* locus in this study, including 11 deletions encompassing the entire *FOXL2* gene, nine deletions located upstream of *FOXL2*, one combined upstream and downstream deletion flanking *FOXL2* and one novel chromosomal translocation involving the *FOXL2* genomic region.

B. Novel structural variants affecting *FOXL2*		

Type of SV	Coordinates (hg38)	Phenotype
Total *FOXL2* del		BPES
Total *FOXL2* del		BPES
Total *FOXL2* del		BPES
Total *FOXL2* del		BPES
Total *FOXL2* del		BPES and cryptorchidism
Total *FOXL2* del	Chr3 (138945645–142436590)	BPES
Total *FOXL2* and *ATR* del	Chr3 (136422338–148529357)	BPES
Total *FOXL2* and *ATR* del		BPES
Total *FOXL2* and *ATR* del	Chr3 (129785551–148834776)	BPES
Total *FOXL2* del		BPES
Total *FOXL2* del		BPES
Deletion upstream of *FOXL2*	*PISRT1*	BPES
Deletion upstream of *FOXL2*	Chr3 (139230577–139234837)	BPES
Deletion upstream of *FOXL2*	Chr3 (139230577–139236822)	BPES
Deletion upstream and downstream of *FOXL2*	Deletion 1: chr3 (138640184–138819066) Deletion 2: chr3 (139003469–139366791)	BPES
Deletion upstream of *FOXL2*	Chr3 (139170001–139560000)	BPES
Deletion upstream of *FOXL2*	*PISRT1*	BPES
Deletion upstream of *FOXL2*	*PISRT1*	BPES
Deletion upstream of *FOXL2*	*PISRT1*	BPES
Deletion upstream of *FOXL2*	Chr3 (139046001–139519000)	BPES
Deletion upstream of *FOXL2*	Chr3 (139189424–139251840)	BPES
46,XY,t(3,18)(q23;q21.3)		BPES

### 3.2. *FOXL2* Coding Sequence Variants and Their Variant Classification

A total of 752 index patients have been identified with a *FOXL2* coding sequence variant making up 87% (752/864) of the total solved BPES index cases and revealed 54 unique novel variants in the coding region of the *FOXL2* gene. The majority of these index patients are heterozygous for a frameshift variant (39%; 295/752), followed by in‐frame indels (31%; 231/752), missense variants (18%; 137/752), nonsense variants (11%; 82/752) and stop‐loss variants (1%; 7/752) (Supporting Information 1: Table S1, Figure 2).

FIGURE 2(a) In 87% of the BPES patient cohort (*n* = 752), variants were identified in the coding region of *FOXL2*. These include frameshift (39%), in‐frame indel (31%), missense (18%), nonsense (11%) and stop‐loss (1%) variants. (b) Coding *FOXL2* variants are distributed across the entire protein. Missense variants cluster in the forkhead domain, while in‐frame indels predominantly affect the polyalanine tract, both known mutational hotspots of the *FOXL2* region. Novel variants identified in this study are highlighted in yellow.

**Figure:**
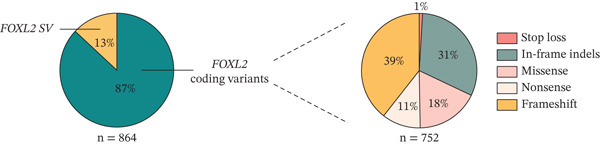
(a)

**Figure:**
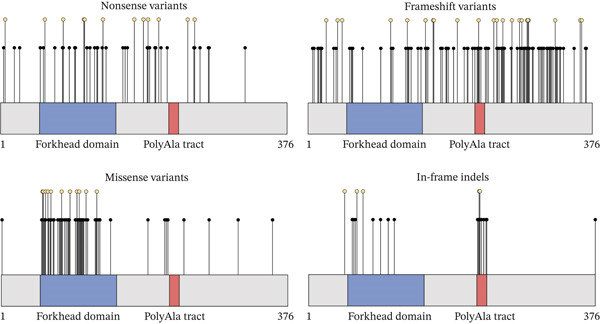
(b)

#### 3.2.1. Frameshift Variants

Our compendium contains 135 unique frameshift variants, including 22 novel variants, of which 24% (32/135) are classified as pathogenic and 76% (103/134) are classified as likely pathogenic. Frameshifts of the *FOXL2* coding region result in either a predicted truncated protein (100/135) or an elongated protein (35/135), leading to loss‐of‐function. Notably, the most recurrent frameshift variants, including c.843_859dup (p.[Pro287Argfs∗75]) (*n* = 66), c.855_871dup (p.[His291Argfs∗71]) (*n* = 38) and c.804dup (p.[Gly269Argfs∗265]) (*n* = 20), are clustered downstream of the polyalanine tract (Supporting Information 1: Table S1, Figure 2).

#### 3.2.2. In‐Frame Indels

In addition to the 23 previously reported unique in‐frame indel variants, we report three novel in‐frame variants located outside the polyalanine tract (c.142_173delinsGCGCT, p.[Lys48_Ser58delinsAlaLeu]; c.191_233delins43, p.[Ala64_Ser78delins15] and c.214_216del, p.[Glu72del]) and two novel in‐frame duplications located within the polyalanine tract (c.666_698dup, p.[Ala224_Ala234dup] and c.701_702ins30, p.[Ala225_Ala234dup]) in this study. Of all unique variants, 32% are classified as pathogenic (9/28) and 68% as likely pathogenic (19/28).

**FIGURE 3:**
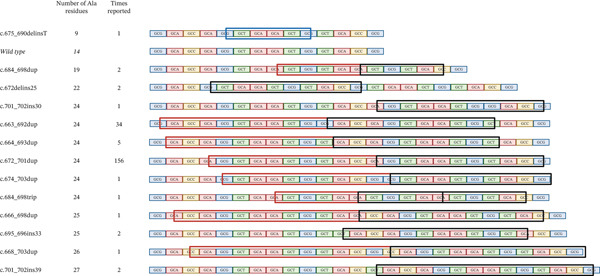
Polyalanine expansions were identified in 24% of the BPES patient cohort, making this the most frequent variant type. In most cases, the polyalanine tract is expanded from 14 to 24 alanine residues. Additionally, one contraction of the polyalanine tract was observed. The blue bar indicates deleted amino acids, the red bar represents the original repeat that was duplicated and the black bar denotes the inserted repeat.

To date, 12 unique duplications and one deletion in the polyalanine region have been described. Polyalanine expansions are the most frequently reported type of in‐frame indels and, to a larger extent, the most frequently reported *FOXL2* variants in BPES, as they are identified in 209 BPES index patients in this large cohort (24%; 209/864). The most prevalent polyalanine expansion is c.672_701 (p.[Ala225_Ala234dup]), observed in at least 156 BPES index cases, including 69 from our patient cohort. Another common polyalanine expansion, c.663_692dup (p.[Ala225_Ala234dup]), has been reported 13 times in literature and is also found in 21 index cases within our patient cohort (Supporting Information 1: Table S1, Figures 2 and 3).

Interestingly, a small polyalanine expansion of 5+ alanine residues (c.684_698dup; p.[Ala230_Ala234dup]) was reported in an Indian, multigenerational family. Heterozygous family members remained (seemingly) unaffected, whereas homozygous individuals had a typical BPES phenotype. One of the homozygous female family members was also diagnosed with POI [78]. Notably, the same variant was identified in an unrelated, sporadic case in a heterozygous state with a typical BPES phenotype [104] (Supporting Information 1: Table S1).

#### 3.2.3. Missense Variants

Next, 84 unique missense variants were found of which the majority are classified as pathogenic (33%; 28/84) or likely pathogenic (61%; 51/84), and 6% as VUS (5/84). Most (*n* = 74) are located in the forkhead domain, which is known to be a mutational hotspot for missense variants in *FOXL2*. We have identified 13 novel missense variants, all located within the forkhead domain (Supporting Information 1: Table S1, Figure 2).

Single nucleotide polymorphisms (SNPs) have previously been reported in the *FOXL2* region, including c.501C > T (p.Phe167=), c.536C > G (p.Ala179Gly), c.647C > T (p.Ala216Val) and c.869C > A (p.Pro290His) [60, 64, 74, 85, 90]. These variants have occasionally been detected in patients with BPES; however, their presence has also been documented in unaffected individuals, and they are catalogued in public SNP databases (e.g., dbSNP: rs7432551, rs61750361 and rs565208053). This suggests that they represent population polymorphisms rather than pathogenic variants. According to ACMG classification criteria, their relatively high allele frequencies in gnomAD, together with consistent *in silico* predictions of benign impact, further support that these variants should be considered (likely) benign.

#### 3.2.4. Nonsense Variants

We identified 51 unique nonsense variants in a total of 82 index patients, of which 12 are novel. All variants were classified as pathogenic (35%; 18/51) or likely pathogenic (65%; 33/51). Most of these variants are observed only once, with the notable exception of the recurrent variant c.655C > T (p.[Gln219∗]), reported 10 times. Another nonsense variant, p.(Trp128∗), has been reported six times, albeit resulting from different nucleotide changes (c.383G > A, c.384G > A and c.384del) (Supporting Information 1: Table S1, Figure 2).

#### 3.2.5. Stop‐Loss Variants

Finally, a total of three unique stop‐loss variants, all classified as likely pathogenic, have been identified in seven BPES index cases. Amongst these, two are novel stop‐loss variants, c.1130G > C (p.(∗377Serext∗31)) and c. 1331A > G (p.(∗377Trpext∗31)), found in two and one BPES index cases, respectively (Supporting Information 1: Table S1, Figure 2).

### 3.3. SVs Affecting the *FOXL2* Region

#### 3.3.1. Copy Number Variants of the *FOXL2* Region

CNVs affecting the *FOXL2* gene were identified in 8% (71/864) of this BPES patient cohort and were detected using MLPA *FOXL2*‐specific probes (Supporting Information 2: Table S2, Figure 4). All but one of these CNVs encompassed the entire *FOXL2* gene. A single case involved a partial *FOXL2* deletion (BPES_10). The *ATR* gene, also covered by the MLPA assay, was codeleted in approximately 23% of all detected deletions. Notably, all reported CNVs exclusively comprised deletions, with no instances of duplications associated with BPES documented to date. Furthermore, literature review revealed an additional 23 cases with *FOXL2* whole gene deletions, albeit lacking specific genomic coordinates. Via DECIPHER, we were able to identify six more patients with BPES harbouring a *FOXL2* (and *ATR*) deletion, with detailed genomic coordinates provided. In‐house, 31 previously reported cases with BPES were molecularly diagnosed using MLPA followed by qPCR and/or microarray‐based copy number analysis, enabling precise delineation of the deletions [18–20, 85]. Amongst the deletions that were further delineated, nearly all extended beyond *FOXL2* to encompass one or more neighbouring genes. Additionally, we report 11 novel deletions spanning the entire *FOXL2* region in this study.

FIGURE 4(a) SVs were identified in 13% of the BPES patient cohort (*n* = 112). These include whole‐gene deletions of *FOXL2* (64%), deletions located up‐ or downstream of *FOXL2* (22%) and translocations (14%). (b) Translocation breakpoints cluster upstream of *FOXL2*, disrupting the regulatory landscape and physically separating the gene from its regulatory elements. Moreover, upstream deletions share an SRO (indicated in orange) of 4.5 kb, encompassing putative *FOXL2* enhancers, including the lncRNA *PISRT1*. The topologically associated domain (TAD) of *FOXL2* is highlighted with a red bar, marking the boundaries of its regulatory domain. Deletions for which no further delineation was performed; bars indicate whether a *FOXL2* or *PISRT1* deletion was detected by MLPA, although the exact genomic coordinates of these deletions remain undetermined.

**Figure:**
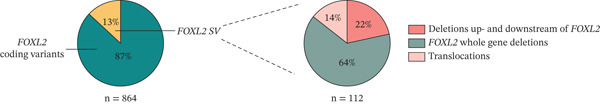
(a)

**Figure:**
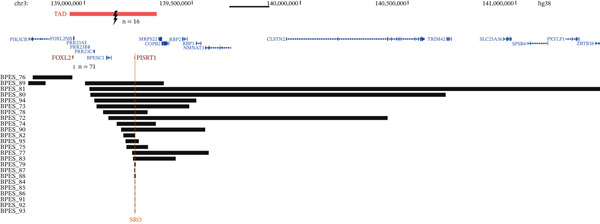
(b)

Deletions disrupting the *FOXL2 cis*‐regulatory region, including the lncRNA *PISRT1*, were identified in 3% (24/864) of genetically solved BPES index patients, ultimately leading to aberrant *FOXL2* expression (Supporting Information 2: Table S2, Figure 4). In‐house, 10 typical patients with BPES harbouring a *cis*‐regulatory deletion could be identified, as previously published by us [18–20, 85]. One of the deletions is located downstream of *FOXL2*, whereas the other nine deletions are located upstream of *FOXL2*. In Bouman *et al*, a noncoding 54‐kb deletion (234 kb upstream of *FOXL2*) is described that encompasses the upstream *cis*‐regulatory region including *PISRT1* in a 9‐month‐old female patient and her father, both displaying a typical BPES phenotype. Based on this regulatory deletion, the shortest region of overlap (SRO) of all noncoding deletions, located upstream of *FOXL2*, could be delineated to a region of 4573 bp (chr3: 139230309–139234882 [Hg38]) [35]. Additionally, we describe 10 cases with BPES in this study with novel deletions upstream of *FOXL2*, all of which include the *PISRT1* region.

Importantly, one exceptional case (BPES_89) initially appeared to involve only a *PISRT1* deletion, as revealed by MLPA. However, subsequent CNV‐sequencing analysis revealed the presence of two distinct deletions, located up‐ and downstream of *FOXL2*, indicating a more complex structural rearrangement of the *FOXL2* locus (Supporting Information 2: Table S2, Figure 4).

Another interesting case described a Chinese family with BPES in which all affected males additionally exhibited polydactyly (BPES_96, Supporting Information 2: Table S2). The causative variant was a deletion within the *FOXL2* promoter overlapping a CRE, and functional luciferase assays demonstrated reduced promoter activity, supporting a regulatory loss‐of‐function mechanism [118].

#### 3.3.2. Translocations

Lastly, cytogenetic rearrangements of 3q23 were found in 2% (16/864) of typical BPES index patients. Fifteen translocations had been described in literature before [4, 58, 75, 94, 106–111, 113–115, 119, 120] (Supporting Information 3: Table S3, Figure 4). Additionally, we identified one novel *de novo* translocation t(3,18)(q23; q21.3) in a male patient with BPES. None of the identified translocations disrupted the *FOXL2* coding sequence itself. Instead, all mapped breakpoints clustered upstream of *FOXL2* within its *cis*‐regulatory domain, consistent with a position‐effect mechanism in which disruption of long‐range enhancers leads to *FOXL2* dysregulation, as previously shown [94, 109, 112, 113, 115].

## 4. Discussion

### 4.1. Biological Relevance

FOXL2 is a crucial transcription factor in both eyelid and ovarian development, but also postnatally FOXL2 remains critical for maintenance of the ovaries. Pathogenic variants in the *FOXL2* gene underlie BPES, associating an eyelid malformation with POI.

In this study, we compiled a comprehensive compendium of (virtually) all reported *FOXL2* genetic defects associated with BPES and of our in‐house patient cohort, encompassing both (non)coding sequence variants and SVs. Across a total of 864 index cases, we distinguished 413 unique genetic defects of the *FOXL2* region, including 76 novel variants.

The majority of *FOXL2* genetic defects are coding sequence variants, as they are identified in 87% of molecularly solved index patients with BPES.

As *FOXL2* is a single‐exon gene, aberrant transcripts are expected to escape nonsense‐mediated decay (NMD). Consequently, null variants, introducing premature stop codons, are predicted to give rise to truncated FOXL2 proteins, missing the forkhead domain and/or the polyalanine tract [85]. In contrast to the wild‐type FOXL2, which displays exclusive nuclear localization, truncated FOXL2 proteins tend to form nuclear aggregates [26, 121, 122].

Missense variants represent 18% of all sequence variants and are predominantly clustered within the forkhead domain, a well‐established mutational hotspot (Figure 2). Alterations in this domain are compromising DNA binding and transcriptional regulation, a mechanism that has been extensively studied previously [30, 122–125].

A second mutational hotspot within the *FOXL2* gene is the polyalanine tract. In 24% of all BPES index patients, an expansion of the polyalanine tract is found, making this the most recurrent *FOXL2* variant associated with typical BPES. The WT polyalanine tract is located between position p.221 and p.234, consists of 14 alanine residues and is strictly conserved amongst mammals [124, 126]. However, its function remains largely unknown. Up to date, 12 unique duplications and one deletion of the polyalanine tract have been reported (Supporting Information 1: Table S1, Figure 3), demonstrating that BPES can be caused by both expansion and contraction of the polyalanine tract, encoded by imperfect trinucleotide repeats. These expansions are most likely caused by slippage of DNA polymerase during replication of these trinucleotide repeats. It was shown previously that these expansions result in the misfolding of the protein and as such impair FOXL2 protein function in a length‐dependent manner, resulting in protein mislocalization from the nucleus to the cytoplasm with cytoplasmic aggregation of the altered FOXL2 protein [126].

The remainder of *FOXL2* genetic defects consisted of deletions affecting the gene (8%) or its *cis*‐regulatory region (3%), as well as upstream translocations separating *FOXL2* from its *cis*‐regulatory domain (2%). These SVs are also expected to result in *FOXL2* loss‐of‐function through reduced or absent gene expression.

Interestingly, these noncoding SVs represent unique natural human models for studying the *cis*‐regulation of *FOXL2*. We have previously delineated the SRO of regulatory deletions to a 7.4‐kb region upstream of *FOXL2* [20]. Recently, a novel deletion has been reported that delineates the SRO even further to a region of 4.5 kb [35], comprising a cluster of candidate CREs and a lncRNA *PISRT1*. Interestingly, the SRO delineated in human cases with BPES has its counterpart in a natural animal model, namely the Polled Intersex Syndrome (PIS) goat [127–129]. First, the syntenic region affected by a complex noncoding SV in the Polled Intersex goat overlaps with the 4.5‐kb SRO delineated in human BPES. Second, the Polled Intersex phenotype is characterized by the dominant absence of horns in both sexes and recessive sex reversal in XX animals. Another animal model for aberrant *cis*‐regulation of *FOXL2* is the piggyBac mouse (*Foxl2^PB/PB^*), caused by an insertion 160 kb upstream of *Foxl2*, leading to reduced *Foxl2* expression and causing an eyelid phenotype mimicking BPES [130]. Although the syntenic regions in human, goat and mouse are not ultraconserved, the occurrence of cross‐species noncoding defects leading to similar phenotypes supports the hypothesis of a functional conservation of the upstream *cis*‐regulatory region of *FOXL2*. However, the precise function of this *cis*‐regulatory region has remained elusive so far and requires further investigation.

### 4.2. Clinical and Diagnostic Relevance

The BPES phenotype is characterized by a typical eyelid malformation, including blepharophimosis, ptosis, epicanthus inversus and telecanthus, sometimes associated with POI. The phenotype can be distinguished from other syndromes that share one or more overlapping traits, such as Say‐Barber‐Biesecker variant of Ohdo syndrome (OMIM #603736) or Kaufman oculocerebrofacial syndrome (OMIM #244450), caused by variants in *KAT6B* and *UBE3B*, respectively. Also, one case has been described with loss‐of‐function of NR2F2, resulting in a BPES‐like phenotype [131].

However, to date *FOXL2* remains the only known disease gene underlying typical BPES. Importantly, penetrance for the eyelid phenotype in individuals carrying a heterozygous *FOXL2* variant is considered complete, as it is observed in all molecularly solved BPES patients. The only reported exception is a small *FOXL2* polyalanine expansion of +5 residues behaving as a recessive allele in a consanguineous Indian family [78]. This variant likely represents the milder end of a length‐ and dosage‐dependent pathogenic spectrum of *FOXL2* polyalanine expansions, as it was also found in a heterozygous state in a young, male BPES patient [104].

In contrast, the penetrance of POI in BPES patients overall is considered reduced and age related.

Molecular diagnosis remains necessary to genetically confirm BPES, particularly for clinical decision‐making and accurate genetic counselling. Given the autosomal dominant inheritance pattern, genetic counselling is essential in BPES families, both for recurrence risk assessment and reproductive planning. This updated *FOXL2* variant database, including previously published and novel variants (Supporting Information 1–3: Tables S1–S3), curated according to ACMG/AMP criteria, provides a key tool for clinical laboratories and clinicians to aid interpretation of variants identified in patients with BPES.

Because of the highly specific phenotype and complete penetrance of the eyelid malformation, we propose tailored rule specification of ACMG criterion PP4 for *FOXL2* variant classification in BPES (see also Supporting Information 1: Table S1):

- PP4: when BPES is clinically well described and assessed by a physician (distinguished from nonspecific ptosis).
- PP4 moderate: when the phenotype is explicitly confirmed by an expert ophthalmologist.
- PP4 strong: when, in addition, biological parameters such as FSH and AMH levels support a diagnosis of POI.

This phenotype‐driven refinement will facilitate more accurate and consistent variant classification across diagnostic laboratories. In the (Supporting Information 1: Table S1), we propose additional *FOXL2*‐specific rule specifications for variant classification in BPES.

### 4.3. Genotype–Phenotype Correlations

Previous studies have attempted to define a clear genotype–phenotype correlation in BPES. Truncating variants located upstream of the polyalanine tract tend to be linked to BPES Type I (combining the eyelid malformation with POI), whereas polyalanine expansions appear more frequently associated with BPES Type II (presenting only eyelid malformations) [85, 132]. Missense variants within the forkhead domain have generally been correlated with BPES Type II [123, 132]. However, Li *et al*. suggested that missense variants situated in the central region of the forkhead domain may instead lead to the more severe BPES Type I phenotype [26]. Despite these observations, a consistent genotype–phenotype correlation across *FOXL2* variants has not yet been established, making the prediction of POI risk in young female patients with BPES challenging.

Also, in this study, no clear genotype–phenotype correlations could be established, mainly due to the lack of appropriate clinical follow‐up on fertility and endocrinological outcomes in female patients.

For many variants, only a single index patient has been identified, making it difficult to determine whether these variants are associated with an ovarian phenotype. This uncertainty often stems from the fact that some variants were found only in male patients or in female patients who are still too young to evaluate ovarian (mal)function.

Notably, for several recurrent variants observed in multiple female BPES patients, including c.843_859dup [p.(Pro287Argfs*75)], c.655C>T [p.(Gln219*)], c.157C>T [p.(Gln53*)], c.650C>T [p.(Ser217Phe)] and c.672_701dup30 [p.(Ala225_Ala234dup)] (Supporting Information 1: Table S1), the phenotype ranged from no ovarian involvement to primary or secondary amenorrhea, sometimes leading to infertility, demonstrating that this variability is probably not restricted to a specific variant type. In addition, all variant types and positions appeared capable of causing both BPES combined or not with POI, with varying ages of onset of POI.

We therefore propose that BPES should rather be considered a single condition displaying a continuum ranging from a fully penetrant eyelid phenotype to BPES with POI, which shows age‐related penetrance in affected females. Accordingly, we emphasize that all female BPES patients, irrespective of their underlying variant, should receive appropriate endocrinological and fertility follow‐up to enable timely detection and management of ovarian dysfunction.

### 4.4. Diagnostic Perspective for Patients With BPES

Currently, the diagnostic strategy for BPES relies on targeted *FOXL2* sequencing in patients with a typical eyelid phenotype, complemented by CNV analysis of the coding region and upstream noncoding regulatory region. This approach allows the majority of affected individuals to be molecularly confirmed. Nevertheless, approximately 12% of clinically diagnosed BPES patients remain without a molecular diagnosis [15]. One striking example in our cohort was a multigenerational family of Polynesian descent that remained unsolved for many years. As no female family members had offspring, BPES with POI was suspected. Standard diagnostic testing with targeted Sanger sequencing of the *FOXL2* coding region and MLPA failed to identify a pathogenic variant. Whole‐genome sequencing (WGS) of multiple family members eventually revealed the most recurrent polyalanine expansion, c.672_701dup (p.[Ala225_Ala234dup]) to cosegregate with disease. The earlier negative results were likely explained by allelic drop‐out in tested individuals. As this expansion represents a major fraction of *FOXL2* variants, the case illustrates the limitations of current first‐tier approaches and highlights the need for more specifically targeted methodologies. Bunyan *et al*. described a fluorescent PCR‐based assay for detecting *FOXL2* polyalanine expansions, providing a reliable and cost‐effective solution for identifying polyalanine expansions [31].

Beyond repeat expansions, additional classes of pathogenic variation may contribute to the unresolved fraction of BPES cases. Short‐read and long‐read WGS, as well as optical genome mapping, offer the ability to detect cryptic and complex SVs, including balanced rearrangements and long‐range regulatory disruptions affecting the *FOXL2 cis*‐regulatory landscape, which are not captured by current targeted assays. Moreover, most diagnostic workflows do not specifically interrogate the *FOXL2* promoter, raising the possibility that pathogenic deletions or regulatory alterations in this region remain undetected and may also account for part of the missing heritability.

Together, the integration of repeat‐sensitive assays, long‐read sequencing and genome‐wide SV detection approaches has the potential to substantially improve diagnostic yield, reduce the proportion of unsolved BPES cases and further refine the understanding of *FOXL2 cis*‐regulatory mechanisms, ultimately improving patient care and genetic counselling.

## 5. Conclusion

In this study, we present a comprehensive overview and ACMG/AMP‐based classification of virtually all reported *FOXL2* variants associated with BPES up to December 2024 and expand the variant spectrum by reporting 76 novel genetic defects. We showed that genotype–phenotype correlations for POI are not straightforward, making prediction of POI risk in young female patients with BPES particularly challenging. This is partly due to the lack of appropriate longitudinal clinical follow‐up, which is particularly relevant for prepubertal female patients. Here, we also propose that BPES should not be subclassified in two distinct subtypes but rather as a condition with a fully penetrant eyelid malformation, consistently present in all patients, with reduced penetrance and variable expressivity of POI in affected females. Looking forward, improved diagnostic strategies, including (targeted) long‐read sequencing and optical genome mapping to resolve variants in the polyalanine tract and short‐read and long‐read WGS to capture complex SVs and noncoding alterations, will be essential to increase the diagnostic yield and ultimately improve precision medicine in BPES.

## Author Contributions

**Charlotte Matton:** conceptualization, data curation, formal analysis, investigation, visualization, writing of the original draft. **Julie Van De Velde:** data curation, formal analysis, investigation, review and editing. **Marieke De Bruyne:** data curation, formal analysis, review and editing. **Stijn Van De Sompele:** data curation, formal analysis, review and editing. **Sally Hooghe:** data curation, formal analysis, review and editing. **Miriam Bauwens:** review and editing. **Hannes Syryn:** review and editing. **Eva D′haene:** review and editing. **Annelies Dheedene:** formal analysis, review and editing. **Martine Cools:** review and editing. **Wendy Watkins:** formal analysis, review and editing. **Shoko Komatsuzaki:** formal analysis, resources, review and editing. **Ewelina Preizner-Rzucidło:** formal analysis, resources, review and editing. **Alison Ross:** formal analysis, resources, review and editing. **Christine Armstrong:** data curation, formal analysis, review and editing. **David J. Bunyan:** formal analysis, resources, review and editing. **Andrew Shelling:** formal analysis, resources, review and editing. **Andrea L. Vincent:** formal analysis, resources, review and editing. **Sascha Vermeer:** formal analysis, resources, review and editing. **Hannah Verdin:** data curation, formal analysis, review and editing. **Elfride De Baere:** conceptualization, funding acquisition, supervision, review and editing.

## Funding

This research was supported by the Research Foundation Flanders (FWO/11Q3D24N to C.M. and 1802220N to E.D.B.).

## Conflicts of Interest

The authors declare no conflicts of interest.

## Supporting Information

Additional supporting information can be found online in the Supporting Information section.

## Supporting information


**Supporting Information 1** Table S1: All identified frameshift, nonsense, missense, in‐frame indel, and stop‐loss variants within the *FOXL2* coding region that are associated with a blepharophimosis, ptosis, epicanthus inversus syndrome (BPES) phenotype.For each variant, the table provides the genomic coordinates, predicted protein effect, and variant classification according to the ACMG/AMP framework. The applied evidence criteria and corresponding arguments supporting each classification are also included.

**Supporting Information 2** Table S2: Overview of all structural variants (deletions) identified in the *FOXL2* locus and its flanking regions, including intragenic *FOXL2* deletions, combined *FOXL2*‐*ATR* deletions, and deletions located up‐ or downstream of *FOXL2*, including genomic coordinates (when available) and the associated phenotype.

**Supporting Information 3** Table S3: Overview of all translocations that disrupt or reposition the *FOXL2* locus and are associated with a blepharophimosis, ptosis, epicanthus inversus syndrome (BPES) phenotype. These data highlight the contribution of structural chromosomal rearrangements to BPES and illustrate how disruption of *FOXL2* or its regulatory landscape can lead to variable or more severe clinical manifestations.

**Supporting Information 4** Table S4: Overview of the primers that are used for Sanger sequencing in BPES, covering the entire *FOXL2* gene. Supporting Information explains how genetic variants were classified using an ACMG/AMP‐based framework that considers seven types of evidence (population, genotype/phenotype, databases, computational, functional, segregation and allelic data) to determine pathogenicity.

## Data Availability

The data that support the findings of this study are available in the Supporting Information of this article.

## References

[1] Beysen D. , De Paepe A. , and De Baere E. , FOXL2 mutations and Genomic Rearrangements in BPES, Human Mutation. (2009) 30, no. 2, 158–169, 10.1002/humu.20807, 2-s2.0-59749101082, 18726931. 18726931

[2] Tucker E. J. , The Genetics and Biology of FOXL2, Sexual Development. (2022) 16, no. 2-3, 184–193, 10.1159/000519836. 34727551

[3] Cocquet J. , De Baere E. , Gareil M. , Pannetier M. , Xia X. , Fellous M. , and Veitia R. A. , Structure, Evolution and Expression of the FOXL2 Transcription Unit, Cytogenetic and Genome Research. (2003) 101, 206–211, 10.1159/000074338, 2-s2.0-0346058365. 14684984

[4] Crisponi L. , Deiana M. , Loi A. , Chiappe F. , Uda M. , Amati P. , Bisceglia L. , Zelante L. , Nagaraja R. , Porcu S. , Serafina Ristaldi M. , Marzella R. , Rocchi M. , Nicolino M. , Lienhardt-Roussie A. , Nivelon A. , Verloes A. , Schlessinger D. , Gasparini P. , Bonneau D. , Cao A. , and Pilia G. , The Putative Forkhead Transcription Factor FOXL2 Is Mutated in Blepharophimosis/Ptosis/Epicanthus Inversus Syndrome, Nature Genetics. (2001) 27, 159–166, 10.1038/84781, 2-s2.0-0035131812. 11175783

[5] De Baere E. , Dixon M. J. , Small K. W. , Jabs E. W. , Leroy B. P. , Devriendt K. , Gillerot Y. , Mortier G. , Meire F. , Van Maldergem L. , Courtens W. , Hjalgrim H. , Huang S. , Liebaers I. , Van Regemorter N. , Touraine P. , Praphanphoj V. , Verloes A. , Udar N. , Yellore V. , Chalukya M. , Yelchits S. , De Paepe A. , Kuttenn F. , Fellous M. , Veitia R. , and Messiaen L. , Spectrum of FOXL2 Gene Mutations in Blepharophimosis-Ptosis-Epicanthus Inversus (BPES) Families Demonstrates a Genotype–Phenotype Correlation, Human Molecular Genetics. (2001) 10, 1591–1600, 10.1093/HMG/10.15.1591. 11468277

[6] Lehmann O. J. , Sowden J. C. , Carlsson P. , Jordan T. , and Bhattacharya S. S. , Fox′s in Development and Disease, Trends in Genetics. (2003) 19, 339–344, 10.1016/S0168-9525(03)00111-2, 2-s2.0-0037866671. 12801727

[7] Zhang Y. , Kao W. W. Y. , Pelosi E. , Schlessinger D. , and Liu C. Y. , Notch Gain of Function in Mouse Periocular Mesenchyme Downregulates FoxL2 and Impairs Eyelid Levator Muscle Formation, Leading to Congenital Blepharophimosis, Journal of Cell Science. (2011) 124, no. 15, 2561–2572, 10.1242/JCS.085001, 2-s2.0-79961151863, 21730020. 21730020 PMC3138700

[8] Schmidt D. , Ovitt C. E. , Anlag K. , Fehsenfeld S. , Gredsted L. , Treier A. C. , and Treier M. , The Murine Winged-Helix Transcription Factor Foxl 2 Is Required for Granulosa Cell Differentiation and Ovary Maintenance, Development. (2004) 131, no. 4, 933–942, 10.1242/DEV.00969, 2-s2.0-1342327343, 14736745. 14736745

[9] Uda M. , Ottolenghi C. , Crisponi L. , Garcia J. E. , Deiana M. , Kimber W. , Forabosco A. , Cao A. , Schlessinger D. , and Pilia G. , Foxl 2 Disruption Causes Mouse Ovarian Failure by Pervasive Blockage of Follicle Development, Human Molecular Genetics. (2004) 13, no. 11, 1171–1181, 10.1093/HMG/DDH124, 2-s2.0-2942750361, 15056605. 15056605

[10] Ellsworth B. S. , Egashira N. , Haller J. L. , Butts D. L. , Cocquet J. , Clay C. M. , Osamura R. Y. , and Camper S. A. , FOXL2 in the Pituitary: Molecular, Genetic, and Developmental Analysis, Molecular Endocrinology. (2006) 20, no. 11, 2796–2805, 10.1210/ME.2005-0303, 2-s2.0-33751528497, 16840539. 16840539

[11] Castets S. , Roucher-Boulez F. , Saveanu A. , Mallet-Motak D. , Chabre O. , Amati-Bonneau P. , Bonneau D. , Girardin C. , Morel Y. , Villanueva C. , Brue T. , Reynaud R. , and Nicolino M. , Hypopituitarism in Patients With Blepharophimosis and FOXL2 Mutations, Hormone Research in Paediatrics. (2020) 93, no. 1, 30–39, 10.1159/000507249, 32454486. 32454486

[12] Governini L. , Carrarelli P. , Rocha A. L. L. , De Leo V. , Luddi A. , Arcuri F. , Piomboni P. , Chapron C. , Bilezikjian L. M. , and Petraglia F. , FOXL2 in Human Endometrium: Hyperexpressed in Endometriosis, Reproductive Sciences. (2014) 21, no. 10, 1249–1255, 10.1177/1933719114522549, 2-s2.0-84905255410, 24520083. 24520083

[13] Zhang B. , Li S. J. , Yuan H. , Cong S. S. , Zhao S. J. , and Yang X. J. , FOXL2 Knockdown Inhibits the Progression of Endometriosis, American Journal of Reproductive Immunology. (2025) 93, no. 1, e70043, 10.1111/aji.70043, 39776079. 39776079

[14] Uhlenhaut N. H. , Jakob S. , Anlag K. , Eisenberger T. , Sekido R. , Kress J. , Treier A. C. , Klugmann C. , Klasen C. , Holter N. I. , Riethmacher D. , Schütz G. , Cooney A. J. , Lovell-Badge R. , and Treier M. , Somatic Sex Reprogramming of Adult Ovaries to Testes by FOXL2 Ablation, Cell. (2009) 139, no. 6, 1130–1142, 10.1016/J.CELL.2009.11.021, 2-s2.0-71149095052, 20005806. 20005806

[15] Verdin H. , Matton C. , and De Baere E. , Blepharophimosis, Ptosis, and Epicanthus Inversus Syndrome, 2022, GeneReviews. 20301614

[16] Owens N. , Hadley R. C. , and Kloepfer H. W. , Hereditary Blepharophimosis, Ptosis, and Epicanthus Inversus, Journal of the International College of Surgeons. (1960) 33, 558–574, 14429566. 14429566

[17] Shah S. P. , Köbel M. , Senz J. , Morin R. D. , Clarke B. A. , Wiegand K. C. , Leung G. , Zayed A. , Mehl E. , Kalloger S. E. , Sun M. , Giuliany R. , Yorida E. , Jones S. , Varhol R. , Swenerton K. D. , Miller D. , Clement P. B. , Crane C. , Madore J. , Provencher D. , Leung P. , DeFazio A. , Khattra J. , Turashvili G. , Zhao Y. , Zeng T. , Glover J. N. M. , Vanderhyden B. , Zhao C. , Parkinson C. A. , Jimenez-Linan M. , Bowtell D. D. L. , Mes-Masson A.-M. , Brenton J. D. , Aparicio S. A. , Boyd N. , Hirst M. , Gilks C. B. , Marra M. , and Huntsman D. G. , Mutation of FOXL2in Granulosa-Cell Tumors of the Ovary, New England Journal of Medicine. (2009) 360, no. 26, 2719–2729, 10.1056/NEJMoa0902542, 2-s2.0-67649406102. 19516027

[18] D′Haene B. , Nevado J. , Pugeat M. , Pierquin G. , Lowry R. B. , Reardon W. , Delicado A. , García-Miñaur S. , Palomares M. , Courtens W. , Stefanova M. , Wallace S. , Watkins W. , Shelling A. N. , Wieczorek D. , Veitia R. A. , De Paepe A. , Lapunzina P. , and De Baere E. , FOXL2 Copy Number Changes in the Molecular Pathogenesis of BPES: Unique Cohort of 17 Deletions, Human Mutation. (2010) 31, no. 5, E1332–E1347, 10.1002/HUMU.21233, 2-s2.0-77951859405, 20232352. 20232352

[19] Beysen D. , Raes J. , Leroy B. P. , Lucassen A. , Yates J. R. W. , Clayton-Smith J. , Ilyina H. , Sklower Brooks S. , Christin-Maitre S. , Fellous M. , Fryns J. P. , Kim J. R. , Lapunzina P. , Lemyre E. , Meire F. , Messiaen L. M. , Oley C. , Splitt M. , Thomson J. , Van De Peer Y. , Veitia R. A. , De Paepe A. , and De Baere E. , Deletions Involving Long-Range Conserved Nongenic Sequences Upstream and Downstream of FOXL2 as a Novel Disease-Causing Mechanism in Blepharophimosis Syndrome, American Journal of Human Genetics. (2005) 77, no. 2, 205–218, 10.1086/432083, 2-s2.0-22544447060, 15962237. 15962237 PMC1224524

[20] Verdin H. , D′haene B. , Beysen D. , Novikova Y. , Menten B. , Sante T. , Lapunzina P. , Nevado J. , Carvalho C. M. B. , Lupski J. R. , and de Baere E. , Microhomology-Mediated Mechanisms Underlie Non-Recurrent Disease-Causing Microdeletions of the FOXL2 Gene or Its Regulatory Domain, PLOS Genetics. (2013) 9, no. 3, e1003358, 10.1371/JOURNAL.PGEN.1003358, 2-s2.0-84875980851, 23516377. 23516377 PMC3597517

[21] Richards S. , Aziz N. , Bale S. , Bick D. , Das S. , Gastier-Foster J. , Grody W. , Hegde M. , Lyon E. , Spector E. , Voelkerding K. , Rehm H. L. , and ACMG Laboratory Quality Assurance Committee , Standards and Guidelines for the Interpretation of Sequence Variants: A Joint Consensus Recommendation of the American College of Medical Genetics and Genomics and the Association for Molecular Pathology, Genetics in Medicine. (2015) 17, no. 5, 405–424, 10.1038/gim.2015.30, 2-s2.0-84928209346, 25741868. 25741868 PMC4544753

[22] Tavtigian S. V. , Greenblatt M. S. , Harrison S. M. , Nussbaum R. L. , Prabhu S. A. , Boucher K. M. , and Biesecker L. G. , Modeling the ACMG/AMP Variant Classification Guidelines as a Bayesian Classification Framework, Genetics in Medicine. (2018) 20, no. 9, 1054–1060, 10.1038/gim.2017.210, 2-s2.0-85049273706, 29300386. 29300386 PMC6336098

[23] Hongbo W. and Zhang Z. , A Case Report of Primary Infertility With BPES Syndrome With FOXL2 Gene Mutation and PADI6 Gene Mutation, Journal of Clinical Medicine & Surgery; Case Report. (2022) 1, no. 1, 1–9, 10.56439/JCMSR.2022.1101.

[24] Meng T. , Zhang W. , Zhang R. , Li J. , Gao Y. , Qin Y. , and Jiao X. , Ovarian Reserve and ART Outcomes in Blepharophimosis-Ptosis-Epicanthus Inversus Syndrome Patients With FOXL2 Mutations, Frontiers in Endocrinology. (2022) 13, 829153, 10.3389/fendo.2022.829153, 35574016. 35574016 PMC9097277

[25] Eskenazi S. , Bachelot A. , Hugon-Rodin J. , Plu-Bureau G. , Gompel A. , Catteau-Jonard S. , Molina-Gomes D. , Dewailly D. , Dodé C. , Christin-Maitre S. , and Touraine P. , Next Generation Sequencing Should Be Proposed to Every Woman With “Idiopathic” Primary Ovarian Insufficiency, Journal of the Endocrine Society. (2021) 5, no. 7, 1–10, 10.1210/jendso/bvab032, 34095689. PMC8169040 34095689

[26] Li F. , Chen H. , Wang Y. , Yang J. , Zhou Y. , Song X. , and Fan J. , Functional Studies of Novel FOXL2 Variants in Chinese Families With Blepharophimosis–Ptosis–Epicanthus Inversus Syndrome, Frontiers in Genetics. (2021) 12, 616112, 10.3389/FGENE.2021.616112/BIBTEX. 33796131 PMC8007913

[27] Rong W.-N. , Yang W. , Yuan S.-Q. , and Sheng X.-L. , Identification of a Novel FOXL2 Mutation in a Fourth-Generation Chinese Family With Blepharophimosis-Ptosis-Epicanthus Inversus Syndrome, International Journal of Ophthalmology. (2021) 14, no. 4, 504–509, 10.18240/ijo.2021.04.04, 33875939. 33875939 PMC8025160

[28] Zheng B. , Seltzsam S. , Wang C. , Schierbaum L. , Schneider S. , Wu C. H. W. , Dai R. , Connaughton D. M. , Nakayama M. , Mann N. , Stajic N. , Mane S. , Bauer S. B. , Tasic V. , Nam H. J. , Shril S. , and Hildebrandt F. , Whole-Exome Sequencing Identifies FOXL2, FOXA2 and FOXA3 as Candidate Genes for Monogenic Congenital Anomalies of the Kidneys and Urinary Tract, Nephrology Dialysis Transplantation. (2022) 37, no. 10, 1833–1843, 10.1093/NDT/GFAB253, 34473308. PMC9755999 34473308

[29] Wang S. , Ge S. , and Zhuang A. , A Novel Forkhead Box L2 Missense Mutation, c.1068G>C, in a Chinese Family With Blepharophimosis/Ptosis/Epicanthus Inversus Syndrome, Journal of Craniofacial Surgery. (2022) 33, no. 3, E238–E240, 10.1097/SCS.0000000000008042, 34374675. 34374675

[30] Hu J. , Ke H. , Luo W. , Yang Y. , Liu H. , Li G. , Qin Y. , Ma J. , and Zhao S. , A Novel FOXL2 Mutation in Two Infertile Patients With Blepharophimosis–Ptosis–Epicanthus Inversus Syndrome, Journal of Assisted Reproduction and Genetics. (2020) 37, no. 1, 223–229, 10.1007/s10815-019-01651-2, 31823134. 31823134 PMC7000634

[31] Bunyan D. J. and Thomas N. S. , Screening of a Large Cohort Of Blepharophimosis, Ptosis, and Epicanthus Inversus Syndrome Patients Reveals a Very Strong Paternal Inheritance Bias and a Wide Spectrum of Novel FOXL2 Mutations, European Journal of Medical Genetics. (2019) 62, no. 7, 103668, 10.1016/J.EJMG.2019.05.007, 2-s2.0-85065389111. 31077882

[32] Bertini V. , Valetto A. , Baldinotti F. , Azzarà A. , Cambi F. , Toschi B. , Giacomina A. , Gatti G. L. , Gana S. , Caligo M. A. , and Bertelloni S. , Blepharophimosis, Ptosis, Epicanthus Inversus Syndrome: New Report With a 197-kb Deletion Upstream of FOXL2 and Review of the Literature, Molecular Syndromology. (2019) 10, 147–153, 10.1159/000497092, 2-s2.0-85063380620. 31191203 PMC6528085

[33] Chacón-Camacho O. F. , Salgado-Medina A. , Alcaraz-Lares N. , López-Moreno D. , Barragán-Arévalo T. , Nava-Castañeda A. , Rodríguez-Uribe G. , Lieberman E. , Rodríguez-Cabrera L. , González-Del Angel A. , Borbolla A. M. , Fernández-Hernández L. , Graue-Hernández E. O. , and Zenteno J. C. , Clinical Characterization and Identification of Five Novel FOXL2 Pathogenic Variants in a Cohort of 12 Mexican Subjects With the Syndrome of Blepharophimosis-Ptosis-Epicanthus Inversus, Gene. (2019) 706, 62–68, 10.1016/J.GENE.2019.04.073, 2-s2.0-85065077602. 31048069

[34] Grzechocińska B. , Warzecha D. , Wypchło M. , Ploski R. , and Wielgoś M. , Premature ovarian insufficiency as a variable feature of blepharophimosis, ptosis, and epicanthus inversus syndrome associated with c.223C > T p.(Leu75Phe) FOXL2 mutation: a case report, BMC Medical Genetics. (2019) 20, no. 1, 10.1186/S12881-019-0865-0, 2-s2.0-85070901554. PMC6670140 31366388

[35] Bouman A. , Van Haelst M. , and Van Spaendonk R. , Blepharophimosis-Ptosis-Epicanthus Inversus Syndrome Caused by a 54-kb Microdeletion in a FOXL2 cis-Regulatory Element, Clinical Dysmorphology. (2018) 27, no. 2, 58–62, 10.1097/MCD.0000000000000216, 2-s2.0-85044581625, 29481440. 29481440

[36] Yang X.-W. , He W.-B. , Gong F. , Li W. , Li X.-R. , Zhong C.-G. , Lu G.-X. , Lin G. , Du J. , Tan Y.-Q. , and Yue-Qiu T. C. , Novel FOXL2 Mutations Cause Blepharophimosis-Ptosis-Epicanthus Inversus Syndrome With Premature Ovarian Insufficiency, Molecular Genetics & Genomic Medicine. (2018) 6, no. 2, 261–267, 10.1002/mgg3.366, 2-s2.0-85045524323. 29378385 PMC5902393

[37] Zhou L. , Wang J. , and Wang T. , Functional Study on New FOXL2 Mutations Found in Chinese Patients With Blepharophimosis, Ptosis, Epicanthus Inversus Syndrome, BMC Medical Genetics. (2018) 19, 10.1186/S12881-018-0631-8/FIGURES/4. PMC6053710 30029625

[38] Cheng H. , Wang T. , Wang G. , Wang J. , Shen L. , Han M. , Yang S. , Shi Y. , Wang W. , and Li H. , Analysis of FOXL2 Gene Mutation and Genotype-Phenotype Correlation in a Chinese Pedigree Affected With Blepharophimosis-Ptosis-Epicanthus Inversus Syndrome, Zhonghua Yi Xue Yi Chuan Xue Za Zhi. (2018) 35, no. 4, 515–517, 10.3760/CMA.J.ISSN.1003-9406.2018.04.012, 2-s2.0-85053709534, 30098246. 30098246

[39] Li F. , Chai P. , Fan J. , Wang X. , Lu W. , Li J. , Ge S. , Jia R. , Zhang H. , and Fan X. , A Novel FOXL2 Mutation Implying Blepharophimosis-Ptosis-Epicanthus Inversus Syndrome Type I, Cellular Physiology and Biochemistry. (2018) 45, no. 1, 203–211, 10.1159/000486358, 2-s2.0-85040719851. 29339661

[40] Li H. and Gu Y. , Genetic and Functional Analyses of Two Missense Mutations in the Transcription FactorFOXL2in Two Chinese Families With Blepharophimosis-Ptosis-Epicanthus Inversus Syndrome, Genetic Testing and Molecular Biomarkers. (2018) 22, no. 10, 585–592, 10.1089/gtmb.2018.0064, 2-s2.0-85055072928. 30234390

[41] Niu B. B. , Tang N. , Xu Q. , and Chai P. W. , Genomic Disruption of FOXL2 in Blepharophimosis-Ptosis-Epicanthus Inversus Syndrome Type 2: A Novel Deletion-Insertion Compound Mutation, Chinese Medical Journal. (2018) 131, no. 19, 2380–2383, 10.4103/0366-6999.241818, 2-s2.0-85054040564, 30246734. 30246734 PMC6166469

[42] Yang L. , Li T. , and Xing Y. , Identification of a Novel FOXL2 Mutation in a Single Family With Both Types of Blepharophimosis-Ptosis-Epicanthus Inversus Syndrome, Molecular Medicine Reports. (2017) 16, no. 4, 5529–5532, 10.3892/mmr.2017.7226, 2-s2.0-85028706776, 28849110. 28849110

[43] Duarte A. F. , Akaishi P. M. S. , de Molfetta G. A. , Chodraui-Filho S. , Cintra M. , Toscano A. , Silva W. A. , and Cruz A. A. V. , Lacrimal Gland Involvement in Blepharophimosis-Ptosis-Epicanthus Inversus Syndrome, Ophthalmology. (2017) 124, no. 3, 399–406, 10.1016/J.OPHTHA.2016.10.028, 2-s2.0-85007486800, 27914838. 27914838

[44] Yang X. , Li W. , Du J. , Yuan S. , He W. , Zhang Q. , Zhong C. , Lu G. , and Tan Y. , Analysis of FOXL2 Gene Mutations in 5 Families Affected With Blepharophimosis, Ptosis and Epicanthus Inversus Syndrome, Zhonghua Yi Xue Yi Chuan Xue Za Zhi. (2017) 34, 342–346, 10.3760/CMA.J.ISSN.1003-9406.2017.03.006, 2-s2.0-85026745275. 28604951

[45] Chai P. , Li F. , Fan J. , Jia R. , and Fan X. , Functional Analysis of a Novel FOXL2 Indel Mutation in Chinese Families With Blepharophimosis-Ptosis-Epicanthus Inversus Syndrome Type I, International Journal of Biological Sciences. (2017) 13, no. 8, 1019–1028, 10.7150/ijbs.19532, 2-s2.0-85026887720, 28924383. 28924383 PMC5599907

[46] Krepelova A. , Simandlova M. , Vlckova M. , Kuthan P. , Vincent A. L. , and Liskova P. , Analysis of FOXL2 Detects Three Novel Mutations and an Atypical Phenotype of Blepharophimosis-Ptosis-Epicanthus Inversus Syndrome, Clinical & Experimental Ophthalmology. (2016) 44, no. 9, 757–762, 10.1111/ceo.12783, 2-s2.0-84978929433. 27283035

[47] Tan H. , Yang P. , Li H. , Pan Q. , Liang D. , and Wu L. , A Novel FOXL2 Mutation in a Chinese Family With Blepharophimosis, Ptosis, Epicanthus Inversus Syndrome, Nature. (2015) 2, 15008, 10.1038/hgv.2015.8, 27081523. PMC4785579 27081523

[48] Xue M. , Zheng J. , Zhou Q. , Fielding Hejtmancik J. , Wang Y. , and Li S. , Novel FOXL2 Mutations in Two Chinese Families With Blepharophimosis-Ptosis-Epicanthus Inversus Syndrome, BMC Medical Genetics. (2015) 16, 10.1186/s12881-015-0217-7, 2-s2.0-84940508268. PMC4593235 26323275

[49] Settas N. , Anapliotou M. , Kanavakis E. , Fryssira H. , Sofocleous C. , Dacou-Voutetakis C. , Chrousos G. P. , and Voutetakis A. , A Novel FOXL2 Gene Mutation and BMP15 Variants in a Woman With Primary Ovarian Insufficiency and Blepharophimosis-Ptosis-Epicanthus Inversus Syndrome, Menopause. (2015) 22, no. 11, 1264–1268, 10.1097/GME.0000000000000473, 2-s2.0-84945926886. 25988799

[50] Nuovo S. , Passeri M. , Di Benedetto E. , Calanchini M. , Meldolesi I. , Di Giacomo M. C. , Petruzzi D. , Piemontese M. R. , Zelante L. , Sangiuolo F. , Novelli G. , Fabbri A. , Brancati F. , Brancati F. , and Fabbri A. , Characterization of Endocrine Features and Genotype-Phenotypes Correlations in Blepharophimosis-Ptosis-Epicanthus Inversus Syndrome Type 1, Journal of Endocrinological Investigation. (2016) 39, 227–233, 10.1007/s40618-015-0334-3, 2-s2.0-84955292935. 26100530

[51] Martinez-Aguayo A. , Poggi H. , Cattani A. , Molina M. , Romeo E. , and Lagos M. , A Novel Insertion in the FOXL2 Gene in a Chilean Patient With Blepharophimosis Ptosis Epicanthus Inversus Syndrome Type I, Journal of Pediatric Endocrinology and Metabolism. (2014) 27, no. 1-2, 181–184, 10.1515/jpem-2013-0219, 2-s2.0-84893757337, 24030029. 24030029

[52] Gulati R. , Verdin H. , Halanaik D. , Bhat B. V. , and De Baere E. , Co-Occurrence of Congenital Hydronephrosis and FOXL2-Associated Blepharophimosis, Ptosis, Epicanthus Inversus Syndrome (BPES), European Journal of Medical Genetics. (2014) 57, no. 10, 576–578, 10.1016/J.EJMG.2014.08.004, 2-s2.0-84908192522, 25192944. 25192944

[53] Jiang H. , Huang X. , Su Z. , Rao L. , Wu S. , Zhang T. , Li K. , Quan Q. , and Zhang K. , Genetic Analysis of the Forkhead Transcriptional Factor 2 Gene in Three Chinese Families With Blepharophimosis Syndrome, Molecular Vision. (2013) 19, 418–423, 23441113. 23441113 PMC3580973

[54] Zhang L. , Wang L. , Han R. , Guan L. , Fan B. , Liu M. , Ying M. , Peng H. , and Li N. , Identification of the Forkhead Transcriptional Factor 2 (FOXL2) Gene Mutations in Four Chinese Families With Blepharophimosis Syndrome, Molecular Vision. (2013) 19, 2298–2305, 24265544. 24265544 PMC3834601

[55] Fan J. , Zhou Y. , Huang X. , Zhang L. , Yao Y. , Song X. , Chen J. , Hu J. , Ge S. , Song H. , and Fan X. , The Combination of Polyalanine Expansion Mutation and a Novel Missense Substitution in Transcription Factor FOXL2 Leads to Different Ovarian Phenotypes in Blepharophimosis–Ptosis–Epicanthus Inversus Syndrome (Bpes) Patients, Human Reproduction. (2012) 27, 3347–3357, 10.1093/HUMREP/DES306, 2-s2.0-84867779178. 22926839

[56] Haghighi A. , Verdin H. , Haghighi-Kakhki H. , Piri N. , Gohari N. S. , and De Baere E. , Missense Mutation Outside the Forkhead Domain of Foxl2 Causes a Severe Form of BPES Type II, Molecular Vision. (2012) 18, 211–218. 22312189 PMC3272052

[57] Zahanova S. , Meaney B. , Łabieniec B. , Verdin H. , De Baere E. , and Nowaczyk M. J. M. , Blepharophimosis-Ptosis-Epicanthus Inversus Syndrome Plus: Deletion 3q22.3q23 in a Patient With Characteristic Facial Features and With Genital Anomalies, Spastic Diplegia, and Speech Delay, Clinical Dysmorphology. (2012) 21, no. 1, 48–52, 10.1097/MCD.0B013E32834977F1, 2-s2.0-83255185104, 21934608. 21934608

[58] González-González C. , García-Hoyos M. , Hernaez Calzón R. , Arroyo Díaz C. , González Fanego C. , Lorda Sánchez I. , and Sánchez-Escribano F. , Microdeletion Found by Array-CGH in Girl with Blepharophimosis Syndrome and Apparently Balanced Translocation t (3;15)(q23;q25), Ophthalmic Genetics. (2012) 33, no. 2, 107–110, 10.3109/13816810.2011.634879, 2-s2.0-84863465452. 22171663

[59] Hu S. , Guo J. , Wang B. , Wang J. , Zhou Z. , Zhou G. , Ding X. , Ma X. , and Qi Y. , Genetic Analysis of the FOXL2 Gene Using Quantitative Real-Time PCR in Chinese Patients With Blepharophimosis-Ptosis-Epicanthus Inversus Syndrome, Molecular Vision. (2011) 17, 436–442, 21321671. 21321671 PMC3038209

[60] Kaur I. , Hussain A. , Naik M. N. , Murthy R. , and Honavar S. G. , Mutation Spectrum of Fork-Head Transcriptional Factor Gene (FOXL2) in Indian Blepharophimosis Ptosis Epicanthus Inversus Syndrome (BPES) Patients, British Journal of Ophthalmology. (2011) 95, 881–886, 10.1136/BJO.2009.177972, 2-s2.0-79956353941. 21325395

[61] Tang S. , Wang X. , Lin L. , Sun Y. , Wang Y. , and Yu H. , Mutation Analysis of the FOXL2 Gene in Chinese Patients With Blepharophimosis-Ptosis-Epicanthus Inversus Syndrome, Mutagenesis. (2006) 21, no. 1, 35–39, 10.1093/MUTAGE/GEI067, 2-s2.0-33344470143. 16394030

[62] Fan J.-Y. , Han B. , Qiao J. , Liu B.-L. , Ji Y.-R. , Ge S.-F. , Song H.-D. , and Fan X.-Q. , Functional Study on a Novel Missense Mutation of the Transcription Factor FOXL2 Causes Blepharophimosis-Ptosis-Epicanthus Inversus Syndrome (BPES), Mutagenesis. (2011) 26, no. 2, 283–289, 10.1093/mutage/geq086, 2-s2.0-79952301355, 21068205. 21068205

[63] Chouchene I. , Derouiche K. , Chaabouni A. , Cherif L. , Amouri A. , Largueche L. , Abdelhak S. , and El Matri L. , Identification of a Novel Mutation in FOXL2 Gene That Leads to Blepharophimosis Ptosis Epicanthus Inversus and Telecanthus Syndrome in a Tunisian Consanguineous Family, Genetic Testing and Molecular Biomarkers. (2010) 14, no. 1, 145–148, 10.1089/gtmb.2009.0091, 2-s2.0-76949086881, 19929410. 19929410

[64] Kraoua L. , Chaabouni M. , Trabelsi M. , Chelly I. , Maazoul F. , Ben Abdallah N. , Boukthir S. , Barsaoui S. , Chaabouni H. , and M′rad R. , FOXL2 Mutations in Tunisian Patients With Blepharophimosis-Ptosis-Epicanthus Inversus Syndrome, Clinical Genetics. (2010) 77, no. 6, 601–603, 10.1111/J.1399-0004.2010.01389.X, 2-s2.0-77953983760, 20236120. 20236120

[65] Lin W.-D. , Chou I. C. , Lee N. C. , Wang C. H. , Hwu W. L. , Lin S. P. , Chao M. C. , Tsai Y. , and Tsai F. J. , FOXL2 Mutations in Taiwanese Patients With Blepharophimosis, Ptosis, Epicanthus Inversus Syndrome, Clinical Chemistry and Laboratory Medicine. (2010) 48, no. 4, 485–488, 10.1515/CCLM.2010.100, 2-s2.0-77949870921, 20184535. 20184535

[66] Zhou Z. , Liang D. , Quan Y. , Xue J. , Li H. , Xia X. , and Wu L. , Deletion and Mutation Analysis to FOXL2 in Blepharophimosis-Ptosis-Epicanthus Inversus Syndrome, Zhonghua Yan Ke Za Zhi. (2010) 46, no. 6, 532–536, 21055199. 21055199

[67] Ni F. , Wen Q. , Wang B. , Zhou S. , Wang J. , Mu Y. , Ma X. , and Cao Y. , Mutation Analysis ofFOXL2gene in Chinese Patients With Premature Ovarian Failure, Gynecological Endocrinology. (2010) 26, no. 4, 246–249, 10.3109/09513590903225358, 2-s2.0-77949454237. 20222838

[68] D′haene B. , Attanasio C. , Beysen D. , Dostie J. , Lemire E. , Bouchard P. , Field M. , Jones K. , Lorenz B. , Menten B. , Buysse K. , Pattyn F. , Friedli M. , Ucla C. , Rossier C. , Wyss C. , Speleman F. , De Paepe A. , Dekker J. , Antonarakis S. E. , and De Baere E. , Disease-Causing 7.4 kb Cis-Regulatory Deletion Disrupting Conserved Non-Coding Sequences and Their Interaction with the FOXL2 Promotor: Implications for Mutation Screening, PLOS Genetics. (2009) 5, e1000522, 10.1371/journal.pgen.1000522, 2-s2.0-67651205709. 19543368 PMC2689649

[69] Méduri G. , Bachelot A. , Duflos C. , Bständig B. , Poirot C. , Genestie C. , Veitia R. , De Baere E. , and Touraine P. , FOXL2 Mutations Lead to Different Ovarian Phenotypes in BPES Patients: Case Report, Human Reproduction. (2010) 25, no. 1, 235–243, 10.1093/HUMREP/DEP355, 2-s2.0-72849121467, 19819892. 19819892

[70] Li D. , Zeng W. , Tao J. , Li S. , Liang C. , Chen X. , Mu W. , Wang X. , Qin Y. , Jie Y. , and Wei W. , Mutations of the Transcription Factor FOXL2 Gene in Chinese Patients With Blepharophimosis-Ptosis-Epicanthus Inversus Syndrome, Genetic Testing and Molecular Biomarkers. (2009) 13, no. 2, 257–268, 10.1089/GTMB.2008.0121, 2-s2.0-67650035454, 19371227. 19371227

[71] Xu Y. , Lei H. , Dong H. , Zhang L. , Qin Q. , Gao J. , Zou Y. , and Yan X. , FOXL2 Gene Mutations and Blepharophimosis–Ptosis–Epicanthus Inversus Syndrome (BPES): A Novel Mutation Detected in a Chinese Family and a Statistic Model for Summarizing Previous Reported Records, Mutagenesis. (2009) 24, no. 5, 447–453, 10.1093/MUTAGE/GEP028, 2-s2.0-71949126279, 19592504. 19592504

[72] Corrêa F. J. S. , Tavares A. B. , Pereira R. W. , and Abrão M. S. , A New FOXL2 Gene Mutation in a Woman With Premature Ovarian Failure and Sporadic Blepharophimosis-Ptosis-Epicanthus Inversus Syndrome, Journal of Fertility and Sterility. (2010) 93, no. 3, 1006.e3–1006.e6, 10.1016/J.FERTNSTERT.2009.08.034, 2-s2.0-75749098036. 19969293

[73] Beysen D. , De Jaegere S. , Amor D. , Bouchard P. , Christin-Maitre S. , Fellous M. , Touraine P. , Grix A. W. , Hennekam R. , Meire F. , Oyen N. , Wilson L. C. , Barel D. , Clayton-Smith J. , De Ravel T. , Decock C. , Delbeke P. , Ensenauer R. , Ebinger F. , Gillessen-Kaesbach G. , Hendriks Y. , Kimonis V. , Laframboise R. , Laissue P. , Leppig K. , Leroy B. P. , Miller D. T. , Mowat D. , Neumann L. , Plomp A. , Van Regemorter N. , Wieczorek D. , Veitia R. A. , De Paepe A. , and De Baere E. , Identification of 34 Novel and 56 Known FOXL2 Mutations in Patients With Blepharophimosis Syndrome, Human Mutation. (2008) 29, E205–E219, 10.1002/HUMU.20819, 2-s2.0-45749097177. 18642388

[74] Nallathambi J. , Laissue P. , Batista F. , Benayoun B. A. , Lesaffre C. , Moumné L. , Pandaranayaka P. E. , Usha K. , Krishnaswamy S. , Sundaresan P. , and Veitia R. A. , Differential Functional Effects of Novel Mutations of the Transcription Factor FOXL2 in BPES Patients, Human Mutation. (2008) 29, no. 8, E123–E131, 10.1002/HUMU.20809, 18484667. 18484667

[75] Tzschach A. , Kelbova C. , Weidensee S. , Peters H. , Ropers H. H. , Ullmann R. , Erdogan F. , Jurkatis J. , Menzel C. , Kalscheuer V. , and Demuth S. , Blepharophimosis-Ptosis-Epicanthus Inversus Syndrome in a Girl With Chromosome Translocation t (2;3)(q33;q23), Ophthalmic Genetics. (2008) 29, no. 1, 37–40, 10.1080/13816810701867615, 2-s2.0-41149158626. 18363172

[76] Leon-Mateos A. , Ginarte M. , Ruiz-Ponte C. , Carracedo A. , and Toribio J. , Blepharophimosis-Ptosis-Epicanthus Inversus Syndrome (BPES), International Journal of Dermatology. (2007) 46, no. 1, 61–63, 10.1111/j.1365-4632.2007.03066.x, 2-s2.0-33845977035. 17214723

[77] Wang J. , Liu J. , and Zhang Q. , FOXL2 Mutations in Chinese Patients With Blepharophimosis-Ptosis-Epicanthus Inversus Syndrome, Molecular Vision. (2007) 13, 108–113, 17277738. 17277738 PMC2533039

[78] Nallathambi J. , Neethirajan G. , Usha K. , Jitendra J. , De Baere E. , and Sundaresan P. , FOXL2 Mutations in Indian Families With Blepharophimosis-Ptosis-Epicanthus Inversus Syndrome, Journal of Genetics. (2007) 86, no. 2, 165–168, 10.1007/S12041-007-0021-Z, 2-s2.0-35548985841, 17968144. 17968144

[79] Or S. F. J. , Tong M. F. T. , Lo F. M. I. , and Lam T. S. S. , Three Novel FOXL2 Gene Mutations in Chinese Patients With Blepharophimosis-Ptosis-Epicanthus Inversus Syndrome, Chinese Medical Journal. (2006) 119, 49–52, 10.1097/00029330-200601010-00009. 16454982

[80] Nallathambi J. , Moumné L. , De Baere E. , Beysen D. , Usha K. , Sundaresan P. , and Veitia R. A. , A Novel Polyalanine Expansion in FOXL2: The first Evidence for a Recessive Form of the Blepharophimosis Syndrome (BPES) Associated With Ovarian Dysfunction, Journal of Human Genetics. (2007) 121, no. 1, 107–112, 10.1007/s00439-006-0276-0, 2-s2.0-33947196237, 17089161. 17089161

[81] Mari F. , Giachino D. , Russo L. , Pilia G. , Ariani F. , Scala E. , Chiappe F. , Sampieri K. , Caporossi A. , Renieri A. , and Lasorella G. , Blepharophimosis, Ptosis, and Epicanthus Inversus Syndrome: Clinical and Molecular Analysis of a Case, Journal of American Association for Pediatric Ophthalmology and Strabismus. (2006) 10, no. 3, 279–280, 10.1016/J.JAAPOS.2006.01.002, 2-s2.0-33745228425, 16814186. 16814186

[82] De Ru M. H. , Gille J. J. P. , Nieuwint A. W. M. , Bijlsma J. B. , Van Der Blij J. F. , and Van Hagen J. M. , Interstitial Deletion in 3q in a Patient With Blepharophimosis-Ptosis-Epicanthus Inversus Syndrome (BPES) and Microcephaly, Mild Mental Retardation and Growth Delay: Clinical Report and Review of the Literature, American Journal of Medical Genetics Part A. (2005) 137, no. 1, 81–87, 10.1002/AJMG.A.30786, 2-s2.0-23344436523, 16015581. 16015581

[83] Raile K. , Stobbe H. , Tröbs R. B. , Kiess W. , and Pfäffle R. , A New Heterozygous Mutation of the FOXL2 Gene Is Associated With a Large Ovarian Cyst and Ovarian Dysfunction in an Adolescent Girl With Blepharophimosis/Ptosis/Epicanthus Inversus Syndrome, European Journal of Endocrinology. (2005) 153, no. 3, 353–358, 10.1530/eje.1.01974, 2-s2.0-26244461936, 16131596. 16131596

[84] Kumar A. , Babu M. , Raghunath A. , and Venkatesh C. P. , Genetic Analysis of a Five Generation Indian Family With BPES: A Novel Missense Mutation (p.Y215C), Molecular Vision. (2004) 10, 445–449, 15257268. 15257268

[85] de Baere E. , Beysen D. , Oley C. , Lorenz B. , Cocquet J. , de Sutter P. , Devriendt K. , Dixon M. , Fellous M. , Fryns J.-P. , Garza A. , Jonsrud C. , Koivisto P. A. , Krause A. , Leroy B. P. , Meire F. , Plomp A. , Maldergem L. V. , de Paepe A. , Veitia R. , and Messiaen L. , FOXL2 and BPES: Mutational Hotspots, Phenotypic Variability, and Revision of the Genotype-Phenotype Correlation, American Journal of Human Genetics. (2003) 72, no. 2, 478–487, 10.1086/346118, 2-s2.0-0037318857, 12529855. 12529855 PMC379240

[86] Udar N. , Yellore V. , Chalukya M. , Yelchits S. , Silva-Garcia R. , Anderson R. , Babul-Hiriji R. , Berhner D. , Brown J. , Callahan A. , Callahan M. , Chitayat D. , Crowley M. , Cytrynbaum C. , Desiletz V. , Dipple K. , Dolliver M. , Fearon J. , Fernandez B. , Fitzbatrick J. , Goldberg R. , Johnson C. , Khaliq A. , Kohn R. , Levin A. , McCann J. , McGillivray B. , Meira L. , Muilengerg A. , Nelson L. , Ochs U. , Pincus D. , Rosengreen S. , Russel K. , Snow J. , Yen M. , and Small K. , Comparative Analysis of the FOXL2 Gene and Characterization of Mutations in BPES Patients, Human Mutation. (2003) 22, no. 3, 222–228, 10.1002/HUMU.10251, 2-s2.0-0041815947, 12938087. 12938087

[87] Cha S. C. , Jang Y. S. , Lee J. H. , Kim H. K. , Kim S. C. , Kim S. , Baek S. H. , Jung W. S. , and Kim J. R. , Mutational Analysis of Forkhead Transcriptional Factor 2 (FOXL2) in Korean Patients With Blepharophimosis-Ptosis-Epicanthus Inversus Syndrome, Clinical Genetics. (2003) 64, no. 6, 485–490, 10.1046/j.1399-0004.2003.00162.x, 2-s2.0-0344740660, 14986827. 14986827

[88] Fokstuen S. , Antonarakis S. E. , and Blouin J. L. , FOXL2-Mutations in Blepharophimosis-Ptosis - Epicanthus Inversus Syndrome (BPES); Challenges for Genetic Counseling in Female Patients, American Journal of Medical Genetics Part A. (2003) 117 A, 143–146, 10.1002/AJMG.A.10024. 12567411

[89] Dollfus H. , Stoetzel C. , Riehm S. , Lahlou Boukoffa W. , Bediard Boulaned F. , Quillet R. , Abu-Eid M. , Speeg-Schatz C. , Francfort J. J. , Flament J. , Veillon F. , and Perrin-Schmitt F. , Sporadic and Familial Blepharophimosis-Ptosis-Epicanthus Inversus Syndrome: FOXL2 Mutation Screen and MRI Study of the Superior Levator Eyelid Muscle, Clinical Genetics. (2003) 63, no. 2, 117–120, 10.1034/J.1399-0004.2003.00011.X, 2-s2.0-0043133777, 12630957. 12630957

[90] Harris S. E. , Chand A. L. , Winship I. M. , Gersak K. , Aittomäki K. , and Shelling A. N. , Identification of Novel Mutations in FOXL2 Associated With Premature Ovarian Failure, Molecular Human Reproduction. (2002) 8, no. 8, 729–733, 10.1093/MOLEHR/8.8.729, 12149404. 12149404

[91] Kosaki K. , Ogata T. , Kosaki R. , Sato S. , and Matsuo N. , A Novel Mutation in the FOXL2 Gene in a Patient With Blepharophimosis Syndrome: Differential Role of the Polyalanine Tract in the Development of the Ovary and the Eyelid, Ophthalmic Genetics. (2002) 23, no. 1, 43–47, 10.1076/OPGE.23.1.43.2202, 2-s2.0-0036210317. 11910558

[92] Ramírez-Castro J. L. , Pineda-Trujillo N. , Valencia A. V. , Muñetón C. M. , Botero O. , Trujillo O. , Vásquez G. , Mora B. E. , Durango N. , Bedoya G. , and Ruiz-Linares A. , Mutations in FOXL2 Underlying BPES (Types 1 and 2) in Colombian Families, American Journal of Medical Genetics. (2002) 113, no. 1, 47–51, 10.1002/ajmg.10741, 2-s2.0-18644370681, 12400065. 12400065

[93] Yamada T. , Hayasaka S. , Matsumoto M. , Budu , Esa T. , Hayasaka Y. , and Endo M. , Heterozygous 17-bp Deletion in the Forkhead Transcription Factor Gene, FOXL2, in a Japanese Family With Blepharophimosis-Ptosis-Epicanthus Inversus Syndrome, Journal of Human Genetics. (2001) 46, no. 12, 733–736, 10.1007/s100380170009, 2-s2.0-0035670452, 11776388. 11776388

[94] De Baere E. , Van Roy N. , Speleman F. , Fukushima Y. , De Paepe A. , and Messiaen L. , Closing in on the BPES Gene on 3q23: Mapping of ade NovoReciprocal Translocation t(3;4)(q23;p15.2) Breakpoint Within a 45-kb Cosmid and Mapping of Three Candidate Genes, RBP1, RBP2, and *β* ^′^-COP, Distal to the Breakpoint, Genomics. (1999) 57, no. 1, 70–78, 10.1006/GENO.1999.5747, 2-s2.0-0033118885, 10191085. 10191085

[95] Boccone L. , Meloni A. , Falchi A. M. , Usai V. , and Cao A. , Blepharophimosis, Ptosis, Epicanthus Inversus Syndrome, a New Case Associated With De Novo Balanced Autosomal Translocation [46,XY, t (3; 7)(q23;q32)], American Journal of Medical Genetics. (1994) 51, 258–259, 10.1002/AJMG.1320510317, 2-s2.0-0028334689. 8074155

[96] Lawson C. T. , Toomes C. , Fryer A. , Carette M. J. M. , Taylor G. M. , Fukushima Y. , and Dixon M. J. , Definition of the Blepharophimosis, Ptosis, Epicanthus Inversus Syndrome Critical Region at Chromosome 3q23 Based on the Analysis of Chromosomal Anomalies, Human Molecular Genetics. (1995) 4, no. 5, 963–967, 10.1093/HMG/4.5.963, 2-s2.0-0029034110, 7633459. 7633459

[97] Wolstenholme J. , Brown J. , Masters K. G. , Wright C. , and English C. J. , Blepharophimosis Sequence and Diaphragmatic Hernia Associated With Interstitial Deletion of Chromosome 3 (46,XY, del (3)(q21q23)), Journal of Medical Genetics. (1994) 31, 647–648, 10.1136/JMG.31.8.647. 7815425 PMC1050030

[98] Costa T. , Pashby R. , Huggins M. , and Teshima I. E. , Deletion 3q in Two Patients With Blepharophimosis-Ptosis-Epicanthus Inversus Syndrome (BPES), Journal of Pediatric Ophthalmology & Strabismus. (1998) 35, no. 5, 271–276, 10.3928/0191-3913-19980901-06, 9782438. 9782438

[99] Jewett T. , Rao P. N. , Weaver R. G. , Stewart W. , Thomas I. T. , and Pettenati M. J. , Blepharophimosis, Ptosis, and Epicanthus Inversus Syndrome (BPES) Associated With Interstitial Deletion of Band 3q22: Review and Gene Assignment to the Interface of Band 3q22.3 and 3q23, American Journal of Medical Genetics. (1993) 47, no. 8, 1147–1150, 10.1002/AJMG.1320470802, 2-s2.0-0027485381, 8291545. 8291545

[100] Ishikiriyama S. and Goto M. , Blepharophimosis Sequence (BPES) and Microcephaly in a Girl With del (3) (q22.2q23): A Putative Gene Responsible for Microcephaly Close to the BPES Gene?, American Journal of Medical Genetics. (1993) 47, no. 4, 487–489, 10.1002/AJMG.1320470411, 2-s2.0-0027429045, 8256811. 8256811

[101] Fujita H. , Meng J. , Kawamura M. , Tozuka N. , Ishii F. , and Tanaka N. , Boy With a Chromosome del (3)(q12q23) and Blepharophimosis Syndrome, American Journal of Medical Genetics. (1992) 44, no. 4, 434–436, 10.1002/AJMG.1320440409, 2-s2.0-0026758081, 1442882. 1442882

[102] Alvarado M. , Bocian M. , Walker A. P. , Opitz J. M. , and Reynolds J. F. , Interstitial Deletion of the Long Arm of Chromosome 3: Case Report, Review, and Definition of a Phenotype, American Journal of Medical Genetics. (1987) 27, no. 4, 781–786, 10.1002/AJMG.1320270406, 2-s2.0-0023634915, 3122568. 3122568

[103] Ye J. , Shi X. , He J. , and Zhang H. , Mutation Analysis of FOXL2 Gene in Chinese Patients With Blepharophimosis-Ptosis-Epicanthus Inversus Syndrome, Zhonghua Yan Ke Za Zhi. (2011) 47, no. 11, 1007–1011, 22336067. 22336067

[104] Wang Y. , Wu Q. , Cao W. , Huang L. , Liu W. , Li C. , and Li N. , Clinical and Genetic Studies of 17 Han Chinese Pedigrees and 31 Sporadic Patients With Blepharophimosis-Ptosis-Epicanthus Inversus Syndrome, Molecular Vision. (2022) 28, 352–358, 36338666. 36338666 PMC9603904

[105] Vincent A. L. , Watkins W. J. , Sloan B. H. , and Shelling A. N. , Blepharophimosis and Bilateral Duane Syndrome Associated With a FOXL2 Mutation, Clinical Genetics. (2005) 68, no. 6, 520–523, 10.1111/J.1399-0004.2005.00527.X, 2-s2.0-28644449823, 16283882. 16283882

[106] Alao M. J. , Lalèyè A. , Lalya F. , Hans C. , Abramovicz M. , Morice-Picard F. , Arveiler B. , Lacombe D. , and Rooryck C. , Blepharophimosis, Ptosis, Epicanthus Inversus Syndrome With Translocation and Deletion at Chromosome 3q23 in a Black African Female, European Journal of Medical Genetics. (2012) 55, no. 11, 630–634, 10.1016/j.ejmg.2012.07.005, 2-s2.0-84867139580, 22906557. 22906557

[107] Al-Awadi S. A. , Naguib K. K. , Farag T. I. , Teebi A. S. , Cuschieri A. , Al-Othman S. A. , and Sundareshan T. S. , Complex Translocation Involving Chromosomes Y, 1, and 3 Resulting in Deletion of Segment 3q23→q25, Journal of Medical. (1986) 23, no. 1, 91–92, 10.1136/JMG.23.1.91, 3950944. PMC1049553 3950944

[108] De Almeida J. C. C. , Neto J. B. G. , Jung M. , Martins R. R. , and Llerna J. C. , Another Example Favouring the Location of BPES at 3q2, Journal of Medical Genetics. (1993) 30, no. 1, 10.1136/JMG.30.1.86, 8481195. PMC1016250 8481195

[109] Crisponi L. , Uda M. , Deiana M. , Loi A. , Nagaraja R. , Chiappe F. , Schlessinger D. , Cao A. , and Pilia G. , FOXL2 Inactivation by a Translocation 171 kb Away: Analysis of 500 kb of Chromosome 3 for Candidate Long-Range Regulatory Sequences, Genomics. (2004) 83, no. 5, 757–764, 10.1016/j.ygeno.2003.11.010, 2-s2.0-1842665188, 15081106. 15081106

[110] De Die-Smulders C. E. M. , Engelen J. J. M. , Donk J. M. , and Fryns J. P. , Further Evidence for the Location of the BPES Gene at 3q2, Journal of Medical Genetics. (1991) 28, no. 10, 10.1136/JMG.28.10.725, 2-s2.0-0025886884, 1941972. PMC1017067 1941972

[111] Fukushima Y. , Wakui K. , Nishida T. , and Ueoka Y. , Blepharophimosis Sequence and De Novo Balanced Autosomal Translocation [46,XY,t(3;4)(q23;p15.2)]: Possible Assignment of the Trait to 3q23, American Journal of Medical Genetics. (1991) 40, 485–487, 10.1002/AJMG.1320400423, 2-s2.0-0025734303. 1746616

[112] Praphanphoj V. , Goodman B. K. , Thomas G. H. , Niel K. M. , Toomes C. , Dixon M. J. , and Geraghty M. T. , Molecular Cytogenetic Evaluation in a Patient With a Translocation (3; 21) Associated With Blepharophimosis, Ptosis, Epicanthus Inversus Syndrome (BPES), Genomics. (2000) 65, no. 1, 67–69, 10.1006/GENO.2000.6157, 2-s2.0-0034176655, 10777667. 10777667

[113] Schlade-Bartusiak K. , Brown L. , Lomax B. , Bruyère H. , Gillan T. , Hamilton S. , McGillivray B. , and Eydoux P. , BPES With Atypical Premature Ovarian Insufficiency, and Evidence of Mitotic Recombination, in a Woman With Trisomy X and a Translocation t (3; 11)(q22.3; q14.1), American Journal of Medical Genetics Part A. (2012) 158 A, no. 9, 2322–2327, 10.1002/AJMG.A.35516, 2-s2.0-84865542985. 22887799

[114] Warburg M. , Bugge M. , and Brøndum-Nielsen K. , Cytogenetic Findings Indicate Heterogeneity in Patients With Blepharophimosis, Epicanthus Inversus, and Developmental Delay, Journal of Medical Genetics. (1995) 32, no. 1, 19–24, 10.1136/JMG.32.1.19, 7897621. 7897621 PMC1050173

[115] Yang Y. , Yang C. , Zhu Y. , Chen H. , Zhao R. , He X. , Tao L. , Wang P. , Zhou L. , Zhao L. , Tu M. , Dong Z. , Chen H. , and Xie Z. , Intragenic and Extragenic Disruptions of FOXL2 Mapped by Whole Genome Low-Coverage Sequencing in Two BPES Families With Chromosome Reciprocal Translocation, Genomics. (2014) 104, no. 3, 170–176, 10.1016/j.ygeno.2014.07.010, 2-s2.0-84922663922, 25086333. 25086333

[116] Yan Y. C. , Zhou L. , and Fan J. C. , Identification and Functional Analyses of a Novel FOXL2 Pathogenic Variant Causing Blepharophimosis, Ptosis, and Epicanthus Inversus Syndrome, International Journal of Ophthalmology. (2023) 16, no. 5, 680–686, 10.18240/IJO.2023.05.02, 37206169. 37206169 PMC10172100

[117] Zhao M. , Meng X. , Wang J. , and Wang T. , Novel FOXL2 Variants in Two Chinese Families With Blepharophimosis, Ptosis, and Epicanthus Inversus Syndrome, Frontiers in Genetics. (2024) 15, 1343411, 10.3389/FGENE.2024.1343411, 38410153. 38410153 PMC10894958

[118] Shen Q. , Zhao X. , Ji Y. , and Chai P. , Deletion of cis-Regulatory Element in FOXL2 Promoter in a Chinese Family of Type II Blepharophimosis-Ptosis-Epicanthus Inversus Syndrome With Polydactyly, Journal of Craniofacial Surgery. (2024) 35, no. 1, 10.1097/SCS.0000000000009801, 37938073. PMC10749674 37938073

[119] Toomes C. and Dixon M. J. , Refinement of a Translocation Breakpoint Associated With Blepharophimosis-Ptosis-Epicanthus Inversus Syndrome to a 280-kb Interval at Chromosome 3q23, Genomics. (1998) 53, 308–314, 10.1006/GENO.1998.5512, 2-s2.0-0032212837. 9799597

[120] Dremsek P. , Schachner A. , Reischer T. , Krampl-Bettelheim E. , Bettelheim D. , Vrabel S. , Delissen Z. , Pfeifer M. , Weil B. , Bajtela R. , Hengstschläger M. , Laccone F. , and Neesen J. , Retrospective Study on the Utility of Optical Genome Mapping as a Follow-Up Method in Genetic Diagnostics, Journal of Medical Genetics. (2025) 62, 89–96, 10.1136/jmg-2024-110265. 39653387 PMC11877032

[121] Moumné L. , Fellous M. , and Veitia R. A. , Deletions in the polyAlanine-containing Transcription Factor FOXL2 Lead to Intranuclear Aggregation, Human Molecular Genetics. (2005) 14, no. 23, 3557–3564, 10.1093/HMG/DDI383, 2-s2.0-28744431913, 16219626. 16219626

[122] Dipietromaria A. , Benayoun B. A. , Todeschini A. L. , Rivals I. , Bazin C. , and Veitia R. A. , Towards a Functional Classification of Pathogenic FOXL2 Mutations Using Transactivation Reporter Systems, Human Molecular Genetics. (2009) 18, no. 17, 3324–3333, 10.1093/HMG/DDP273, 2-s2.0-68749103447, 19515849. 19515849

[123] Beysen D. , Moumné L. , Veitia R. , Peters H. , Leroy B. P. , De Paepe A. , and De Baere E. , Missense Mutations in the Forkhead Domain of FOXL2 Lead to Subcellular Mislocalization, Protein Aggregation and Impaired Transactivation, Human Molecular Genetics. (2008) 17, 2030–2038, 10.1093/HMG/DDN100, 2-s2.0-45749152585. 18372316

[124] Caburet S. , Demarez A. , Moumné L. , Fellous M. , and De Baere E. , A Recurrent Polyalanine Expansion in the Transcription Factor FOXL2 Induces Extensive Nuclear and Cytoplasmic Protein Aggregation, Journal of Medical Genetics. (2004) 41, no. 12, 932–936, 10.1136/jmg.2004.024356, 2-s2.0-10844222804, 15591279. 15591279 PMC1735658

[125] Laissue P. , Lakhal B. , Benayoun B. A. , Dipietromaria A. , Braham R. , Elghezal H. , Philibert P. , Saâd A. , Sultan C. , Fellous M. , and Veitia R. A. , Functional Evidence Implicating FOXL2 in Non-Syndromic Premature Ovarian Failure and in the Regulation of the Transcription Factor OSR2, Journal of Medical Genetics. (2009) 46, 455–457, 10.1136/jmg.2008.065086, 2-s2.0-67650508076. 19429596

[126] Moumné L. , Dipietromaria A. , Batista F. , Kocer A. , Fellous M. , Pailhoux E. , and Veitia R. A. , Differential Aggregation and Functional Impairment Induced by Polyalanine Expansions in FOXL2, a Transcription Factor Involved in Cranio-Facial and Ovarian Development, Human Molecular Genetics. (2008) 17, no. 7, 1010–1019, 10.1093/HMG/DDM373, 2-s2.0-41149124043, 18158309. 18158309

[127] Pailhoux E. , Vigier B. , Chaffaux S. , Servel N. , Taourit S. , Furet J. P. , Fellous M. , Grosclaude F. , Cribiu E. P. , Cotinot C. , and Vaiman D. , A 11.7-kb Deletion Triggers Intersexuality and Polledness in Goats, Nature Genetics. (2001) 29, no. 4, 453–458, 10.1038/ng769, 2-s2.0-0035733723. 11726932

[128] Simon R. , Lischer H. E. L. , Pieńkowska-Schelling A. , Keller I. , Häfliger I. M. , Letko A. , Schelling C. , Lühken G. , and Drögemüller C. , New Genomic Features of the Polled Intersex Syndrome Variant in Goats Unraveled by Long-Read Whole-Genome Sequencing, Animal Genetics. (2020) 51, no. 3, 439–448, 10.1111/AGE.12918, 32060960. 32060960

[129] Vaiman D. , Pailhoux E. , Schibler L. , Oustry A. , Chaffaux S. , Cotinot C. , Fellous M. , and Cribiu E. P. , Genetic Mapping of the Polled/Intersex Locus (PIS) in Goats, Theriogenology. (1997) 47, no. 1, 103–109, 10.1016/S0093-691X(96)00344-5, 2-s2.0-0030623296.

[130] Shi F. , Ding S. , Zhao S. , Han M. , Zhuang Y. , Xu T. , and Wu X. , A piggyBac Insertion Disrupts Foxl2 Expression That Mimics BPES Syndrome in Mice, Human Molecular Genetics. (2014) 23, no. 14, 3792–3800, 10.1093/HMG/DDU092, 2-s2.0-84902968796, 24565867. 24565867

[131] Bashamboo A. , Eozenou C. , Jorgensen A. , Bignon-Topalovic J. , Siffroi J. P. , Hyon C. , Tar A. , Nagy P. , Sólyom J. , Halász Z. , Paye-Jaouen A. , Lambert S. , Rodriguez-Buritica D. , Bertalan R. , Martinerie L. , Rajpert-De Meyts E. , Achermann J. C. , and McElreavey K. , Loss of Function of the Nuclear Receptor NR2F2, Encoding COUP-TF2, Causes Testis Development and Cardiac Defects in 46,XX Children, American Journal of Human Genetics. (2018) 102, no. 3, 487–493, 10.1016/j.ajhg.2018.01.021, 2-s2.0-85042507669, 29478779. 29478779 PMC5985285

[132] Méjécase C. , Nigam C. , Moosajee M. , and Bladen J. C. , The Genetic and Clinical Features of FOXL2-Related Blepharophimosis, Ptosis and Epicanthus Inversus Syndrome, Genes (Basel). (2021) 12, no. 3, 10.3390/GENES12030364, 33806295. PMC7998575 33806295

